# Unraveling the rate-limiting step in microorganisms' mediation of denitrification and phosphorus absorption/transport processes in a highly regulated river-lake system

**DOI:** 10.3389/fmicb.2023.1258659

**Published:** 2023-10-13

**Authors:** Jiewei Ding, Wei Yang, Xinyu Liu, Qingqing Zhao, Weiping Dong, Chuqi Zhang, Haifei Liu, Yanwei Zhao

**Affiliations:** ^1^State Key Laboratory of Water Environment Simulation, School of Environment, Beijing Normal University, Beijing, China; ^2^Shandong Provincial Key Laboratory of Applied Microbiology, Ecology Institute, Qilu University of Technology (Shandong Academy of Sciences), Ji'nan, China

**Keywords:** river-lake ecosystems, nutrient cycling process, biochemical rate-limiting steps, key microorganisms, key genes

## Abstract

River–lake ecosystems are indispensable hubs for water transfers and flow regulation engineering, which have frequent and complex artificial hydrological regulation processes, and the water quality is often unstable. Microorganisms usually affect these systems by driving the nutrient cycling process. Thus, understanding the key biochemical rate-limiting steps under highly regulated conditions was critical for the water quality stability of river–lake ecosystems. This study investigated how the key microorganisms and genes involving nitrogen and phosphorus cycling contributed to the stability of water by combining 16S rRNA and metagenomic sequencing using the Dongping river–lake system as the case study. The results showed that nitrogen and phosphorus concentrations were significantly lower in lake zones than in river inflow and outflow zones (*p* < 0.05). *Pseudomonas, Acinetobacter*, and *Microbacterium* were the key microorganisms associated with nitrate and phosphate removal. These microorganisms contributed to key genes that promote denitrification (*nirB*/*narG*/*narH*/*nasA*) and phosphorus absorption and transport (*pstA*/*pstB*/*pstC*/*pstS*). Partial least squares path modeling (PLS-PM) revealed that environmental factors (especially flow velocity and COD concentration) have a significant negative effect on the key microbial abundance (*p* < 0.001). Our study provides theoretical support for the effective management and protection of water transfer and the regulation function of the river–lake system.

## 1. Introduction

As the most widely distributed ancient community on Earth, microorganisms are characterized by rich species diversity, sensitive responses to environmental cues, and diverse functions (Banerjee et al., 2018). The degradation of pollutants by microorganisms and the biotransformation of nutrients in freshwater systems often drive the system's material circulation and energy flows, thereby contributing to the system's health and stability (Reid et al., 2019; Wu et al., 2021). Microorganisms control geochemical processes in interbasin water transfer and play an irreplaceable role in indicating water quality (Chung et al., 2020; Zhang et al., 2021). Restoring and maintaining a good water microecosystem can promote the improvement of water self-purification capacity, which is conducive to the restoration and improvement of the water ecological environment (Deng et al., 2023). The complex biogeochemical nutrient cycles usually involve multiple steps (Lammel et al., 2015; Zheng et al., 2018; Sankar et al., 2019), in which the functional genes serve as an important medium for the function of microorganisms and can be an important basis for evaluating the functional potential of microbial communities (Djemiel et al., 2022). Wang et al. (2020a) found that in groundwater contaminated with As, *Proteobacteria* can contribute to the expression of As efflux genes *arsB* and *acr*3 to enable microorganisms to display detoxification strategies to pump As out of cells and improve water quality. Wan et al. (2023) have confirmed that in the freshwater system that accepts the addition of foreign organic matter, core microorganisms (e.g., *Rhizobia*) can promote changes in primary productivity by altering the abundance of N_2_-fixation genes, resulting in a healthier and more sustainable freshwater system. In addition, a recent study found that microorganisms can regulate endogenous phosphorus release pollution in natural water bodies through functional genes (*ppk*) that contribute to phosphorus transformation (Zhuo et al., 2023). Hence, understanding the microbial community's composition and functional genes is essential to maintaining the water quality and stability of freshwater ecosystems.

River–lake systems contribute greatly to flood control and storage, water conservation, species conservation, and biodiversity maintenance (Huang et al., 2021; Fernanda et al., 2022). However, the hydrological conditions in river–lake systems were complex, and especially during the period of regulation, flow shocks often led to unstable water quality states (Yang et al., 2017). It is imperative to maintain the water quality and stability of river–lake systems. Many studies have shown that the concentrations of nitrogen and phosphorus are the limiting factors for the water quality stability in river–lake systems (Wang et al., 2020b; Yu et al., 2023). Furthermore, nitrogen and phosphorus are the basic nutrients in the energy flow and material cycle of aquatic ecosystems, and too much or too little would disrupt the ecosystem balance (Zhang et al., 2017; Liang et al., 2021).

Previous studies have found that changes in hydrological conditions (rapid flow, etc.) were critical to shaping the dynamics of freshwater ecosystems (Wiens, 2002; McCluney et al., 2014). Hydrological changes have also been shown to be a major driver for the assembly process of freshwater microbial community aggregation processes and community structure (Pablo Nino-Garcia et al., 2016). Isabwe et al. (2018) also confirmed that the aggregation process of bacterial communities was affected by different hydrological conditions. In the dry season with a low flow rate, the aggregation pattern of bacterial communities was a deterministic process, while in the rainy season with a high flow rate, the bacterial communities were controlled by both stochastic and deterministic processes. In addition, Zhang et al. (2022) found that changes in surface hydrological conditions can lead to differences in microbial community structure and dominant groups, such as the increase of *Actinobacteria* due to low water volume, thus affecting the metabolic activity of microbial communities. Complex hydrological conditions can also affect the composition and potential functional trends of microbial communities (Cheng et al., 2019). Arnon et al. (2007) found that the high flow rate conditions can promote the transfer of chemical substances between water and sediment, provide nutrients for microorganisms, and promote the denitrification process. While the study of Hui et al. (2021) showed that the specific low flow velocity conditions in the flow intersection area would enrich the microorganisms that converted with nitrogen, and these microorganisms could provide *nap* and *narG* genes to effectively promote nitrate removal. Additionally, lakes and rivers have different environmental characteristics, such as organic matter content and pH, which affect the status of certain microbial communities that perform specific functions and consequently affect the stability of water quality (Ren et al., 2017; Tang C. et al., 2020; Crevecoeur et al., 2023). These studies have explored the contribution of microorganisms to the stability of water quality in rivers and lakes and the influence of environmental factors on microbial communities. However, there are still limitations. The key rate-limiting steps for water quality stability in the microbial-driven biochemical cycle are not clear, the ideal microbial communities and genes to improve water quality stability are still unrevealed, and how to promote the formation of these microbial communities and genes through artificial regulation is still not conclusive. These defects are undoubtedly unfavorable to the guidance of water transfer and regulation projects in river–lake systems.

In this study, we surveyed four zones with different hydrological conditions in the Dongping river–lake system. As an important hub of the South-to-North Water Diversion Project, Dongping Lake not only serves the function of water resource regulation but also provides potential drinking water for Beijing and Tianjin. Hence, it is imperative to ensure the water quality stability of the river–lake systems during the regulation period. Our study aimed to (1) identify the differences in physical and chemical properties in different zones of the river–lake system; (2) explore the spatial changes of microbial communities in different zones of the river–lake system; (3) investigate the key biochemical rate-limiting steps of the nitrogen and phosphorus cycles; (4) identify the contribution of key microorganisms and genes to the rate-limiting steps; and (5) determine the environmental variables affecting key microorganisms and genes. Our study will provide suggestions for the coordination of regulation and water quality stability from the perspective of microorganisms and support the long-term sustainable development of river–lake systems.

## 2. Materials and methods

### 2.1. Study area and sample collection

**Figure 1:**
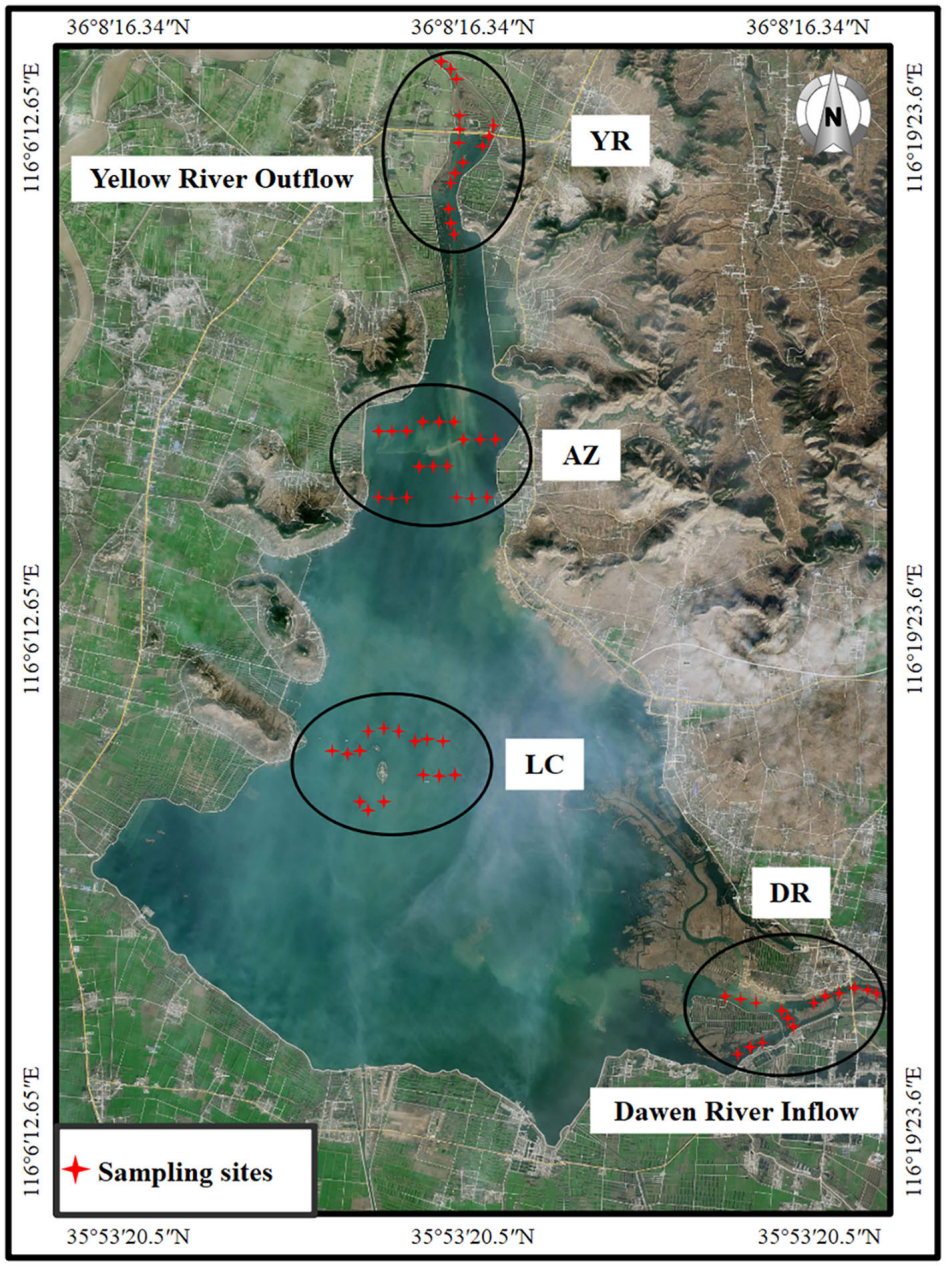
Location map of sampling sites. DR, Dawen River inflow zone; LC, lake center zone; AZ, aquaculture zone; YR, Yellow River outflow zone.

Dongping Lake is an important hub of the world's largest artificial water conservancy project, China's South-to-North Water Diversion Project. It receives its primary inflow from the Dawen River in the east and discharges water to the Yellow River through the two manmade Chenshankou and Qinghemen sluice gates in the north (Figure 1), with a high frequency of regulation (e.g., flow releases and storage). It is a typical river–lake transition area with complex hydrological conditions and drastic changes in water quality (Chen et al., 2014). Especially when the flood was discharged by these artificial gate dams, the huge impact of water flow led to an unstable sedimentary environment and fluctuating water quality.

Based on the strategic position and functional zoning, we divided this lake–river system into four zones: the Dawen River inflow zone (the main inflow river of the lake, DR), the lake center zone (natural ecological protection center area, LC), the aquaculture zone (there are artificial release and naturally raised fish, AZ), and the Yellow River outflow zone (transferring water storage through the sluice gate and providing drinking water source area, YR). The samples were collected in July 2022, during which Dongping Lake is in the artificial flood discharge and regulation period. At each sampling site, we collected five water samples and five sediment samples with triplicate samplings. We collected 1.0-L water samples at a depth of 0.5 m in polyethylene bottles and collected sediment source samples to a depth of 5 cm with a Peterson mud sampler (TC-600BD, Qingdao, China). Samples used to determine physical and chemical properties were immediately sent to a laboratory for testing. Water samples for microbial determination were immediately filtered, and the filter membranes were securely placed at −80°C. Sediment samples were securely placed at −80°C for <48 h until we could extract microbial determination.

### 2.2. Measurement of physicochemical parameters

We measured the water temperature (T), dissolved oxygen (DO), pH, specific conductance of the water (SPC), and the oxidation–reduction potential (ORP) at the study sites using a HACH HQ30d portable measuring instrument (HACH, Loveland, CO, USA). We measured the flow velocity (V) using a Doppler current meter (SF-6526J-21, Beijing, China). We measured the water's transparency (SD) using a Secchi disk *in situ*. For the chemical properties, we measured the chemical oxygen demand (COD), total nitrogen (TN), ammonia nitrogen (${\text{NH}}_{4}^{+}$-N), nitrate nitrogen (${\text{NO}}_{3}^{-}$-N), nitrite nitrogen (${\text{NO}}_{2}^{-}$-N), total phosphorus (TP), and phosphate (${\text{PO}}_{4}^{3-}$) values according to the standard methods defined by China's Ministry of Ecology and Environment (Supplementary Table S1). We measured all environmental parameters three times to mitigate random measurement errors, and we used the average value to represent each sample in our subsequent analyses. All environmental parameters that required laboratory testing were measured within 48 h.

### 2.3. Microbial sequencing and analysis

Microbial bacterial 16s rRNA and metagenomic sequencing were conducted in our study. The 16s rRNA data were used for microbial community structure-related analysis, while metagenomic data were used for microbial gene abundance and key biochemical step analysis. The relevant sequencing processes and analysis methods are shown in the Supplementary Text description of the microbial 16S rRNA and metagenomic sequencing. Our sequencing data has been uploaded to the National Center for Biotechnology Information (NCBL) database. The accession number is SRP457080.

### 2.4. Statistical analyses

The data were analyzed using the SPSS software, version 22.0 (https://www.ibm.com/spss). We used a one-way analysis of variance (ANOVA) with location as the level to test for significant differences. Where the ANOVA results were significant, we used least-significant difference (LSD) tests to identify pairs of values that differed significantly, with significance defined as *p* < 0.05. The independent sample *t*-tests were used for significant differences in topological values for the empirical and random networks (for specific analysis, see Supplementary Text description of the data analysis tools and methods).

## 3. Results

### 3.1. Physical and chemical properties

In the water column (Table 1), the nutrient concentrations of nitrogen (TN, ${\text{NH}}_{4}^{+}$-N, and ${\text{NO}}_{3}^{-}$-N) and phosphorus (TP and ${\text{PO}}_{4}^{3-}$), which were major pollutants in the water environment (Dong et al., 2021), showed the order of YR > DR > AZ > LC. In particular, the values of ${\text{NH}}_{4}^{+}$-N, ${\text{NO}}_{3}^{-}$-N, TP, ${\text{PO}}_{4}^{3-}$ in the YR and DR zones were significantly higher than those of AZ and LC (*p* < 0.05). The concentration of COD also showed significant differences (*p* < 0.05), with the highest concentration in YR (19.0 ± 3.16), followed by DR (17.8 ± 1.17), AZ (16.0 ± 1.40), and LC (15.0 ± 2.00). In addition, although the flow velocity values did not show significant differences in different zones, they also showed a higher trend in the YR and DR. These results showed that the water quality in the lake zone was significantly better than that in the zones where rivers flow in and out (especially in the zones with artificially constructed gates and dams) during the regulation period of the river–lake system. In sediments, the concentration of nitrogen and phosphorus varies in different zones, but the difference was not significant. The COD content (0.58 ± 0.10 mg/g) was highest in the YR, by 0.02, 0.08, and 0.26 mg/g compared with the other zones, but the difference was only significant for the DR.

**Table 1:** Analysis of the physical and chemical properties of water and sediment in the study area.

**Samples**	**Indicators**	**Location**			
		**DR**	**LC**	**AZ**	**YR**
Water	*SD* (cm)	44.800 ± 2.79^b^	81.200 ± 12.54^a^	62.200 ± 14.60^b^	44.800 ± 3.66^b^
	*T* (°C)	27.540 ± 0.27^c^	28.380 ± 0.07^b^	27.100 ± 0.06^c^	30.640 ± 0.82^a^
	*DO* (mg/L)	5.206 ± 0.69^b^	5.588 ± 0.39^b^	7.838 ± 1.07^a^	5.088 ± 0.64^b^
	*SPC* (us/cm^−1^)	722.800 ± 10.13^b^	1,006.000 ± 100.94^a^	1,066.200 ± 66.41^a^	999.600 ± 29.23^a^
	*ORP* (mV)	134.780 ± 2.78^a^	127.600 ± 9.73^a^	112.080 ± 5.75^b^	128.060 ± 8.00^a^
	pH	8.566 ± 0.23^a^	8.534 ± 0.13^a^	8.752 ± 0.16^a^	8.524 ± 0.07^a^
	*V* (m/s)	0.338 ± 0.06^a^	0.280 ± 0.05^a^	0.294 ± 0.05^a^	0.364 ± 0.09^a^
	*D* (m)	2.060 ± 0.56^a^	1.580 ± 0.36^ab^	1.860 ± 0.31^a^	1.070 ± 0.20^b^
	*COD* (mg/L)	17.800 ± 1.17^ab^	15.000 ± 2.00^b^	16.000 ± 1.40^ab^	19.000 ± 3.16^a^
	TN (mg/L)	1.924 ± 0.49^a^	1.810 ± 0.45^a^	1.908 ± 0.50^a^	2.224 ± 0.47^a^
	${\text{NH}}_{4}^{+}$-N (mg/L)	0.146 ± 0.09^a^	0.079 ± 0.06^a^	0.110 ± 0.02^a^	0.366 ± 0.04^a^
	${\text{NO}}_{3}^{-}$-N (mg/L)	0.229 ± 0.04^a^	0.1080 ± 0.05^b^	0.120 ± 0.07^b^	0.350 ± 0.04^a^
	${\text{NO}}_{2}^{-}$-N (mg/L)	0.030 ± 0.02^a^	0.0180 ± 0.01^a^	0.025 ± 0.02^a^	0.017 ± 0.01^a^
	*TP* (mg/L)	0.124 ± 0.04^a^	0.096 ± 0.05^b^	0.088 ± 0.04^b^	0.172 ± 0.06^a^
	${\text{PO}}_{4}^{3-}$ (mg/L)	0.068 ± 0.04^a^	0.056 ± 0.03^b^	0.042 ± 0.02^b^	0.082 ± 0.05^a^
Sediment	*COD* (mg/g)	0.322 ± 0.08^b^	0.500 ± 0.12^a^	0.564 ± 0.11^a^	0.584 ± 0.10^a^
	*TN* (mg/g)	1.444 ± 0.19^a^	1.310 ± 0.26^a^	1.204 ± 0.13^a^	0.848 ± 0.09^a^
	${\text{NH}}_{4}^{+}$-N (mg/g)	0.424 ± 0.06^a^	0.409 ± 0.09^ab^	0.331 ± 0.02^ab^	0.207 ± 0.03^c^
	${\text{NO}}_{3}^{-}$-N (mg/g)	0.172 ± 0.05^a^	0.161 ± 0.06^a^	0.162 ± 0.05^a^	0.193 ± 0.02^a^
	${\text{NO}}_{2}^{-}$-N (mg/g)	0.036 ± 0.01^a^	0.037 ± 0.01^a^	0.040 ± 0.01^a^	0.039 ± 0.01^a^
	*TP* (mg/g)	0.284 ± 0.09^a^	0.276 ± 0.04^a^	0.330 ± 0.05^a^	0.268 ± 0.06^a^
	${\text{PO}}_{4}^{3-}$ (mg/g)	0.208 ± 0.06^a^	0.206 ± 0.03^a^	0.198 ± 0.02^a^	0.192 ± 0.02^a^

Values are mean ± SD. Different letters (a, b, and c) represent significant differences (*p* < 0.05) among sampling sites.

It was worth noting that the TN was strongly and significantly correlated with ${\text{NO}}_{3}^{-}$-N concentration (R = 0.72, *p* < 0.01), as well as TP and ${\text{PO}}_{4}^{3-}$ concentration (R = 0.86, *p* < 0.01) (Supplementary Figure S1). Therefore, the regulation of nitrate and phosphate concentrations was the key to maintaining the steady state of water quality during regulation in this study, and microbial-mediated pathways related to nitrate and phosphate should also be a major focus of this study.

### 3.2. Microbial community structure and composition

**Figure 2:**
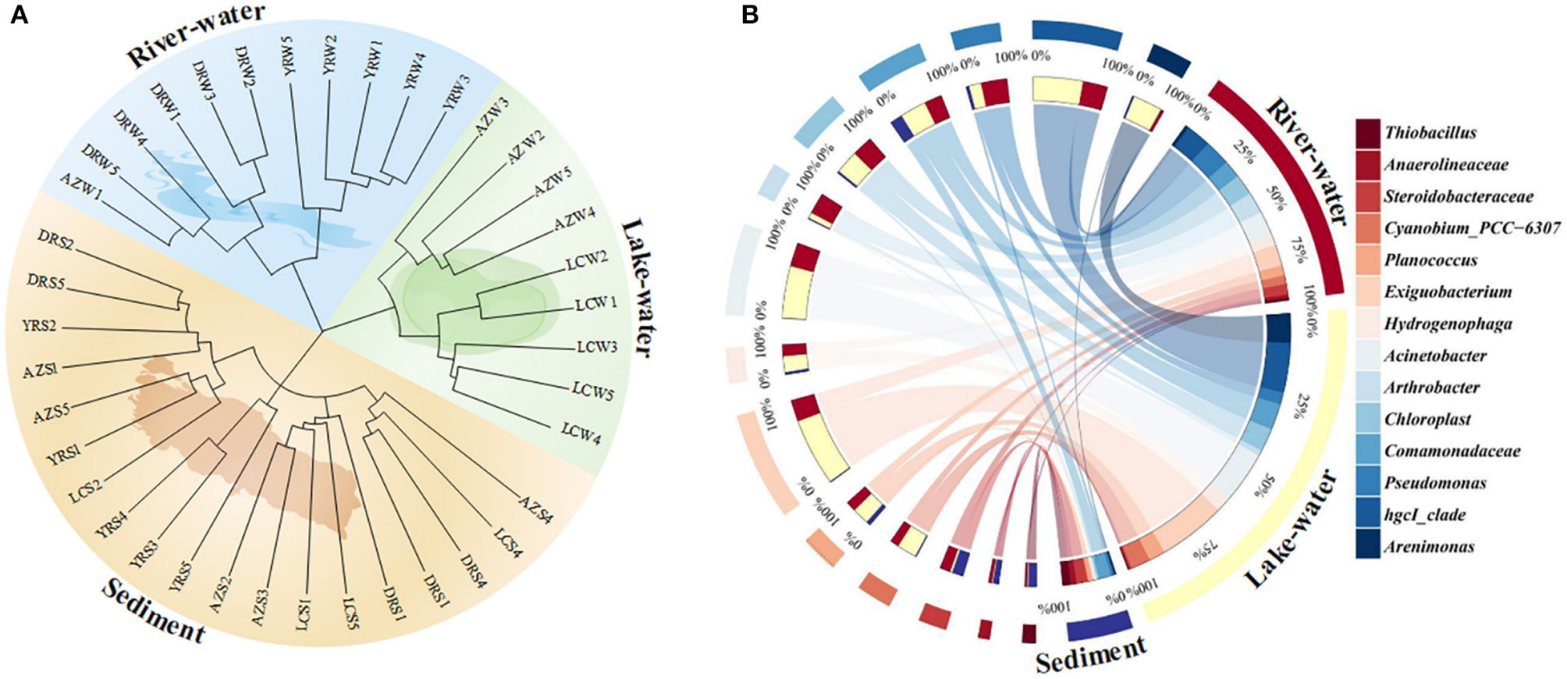
Microbial community structure and composition in the Dongping river-lake system. **(A)** microbial community hierarchical clustering; **(B)** dominant microorganisms at the genus levels in river water, lake water, and sediment. W and S stand for water column and sediment, respectively. Genera that could not be defined were traced back to the family and order level.

Hierarchical clustering and PCoA results showed that all samples in our study can be well classified and clustered into three categories: zones where rivers flow in and out (DRW and YRW), lakes (LCW and AZW), and sediments (DRS, LCS, AZS, and YRS) (Figure 2A; Supplementary Figure S2). In this study, these three categories were defined as river–water, lake–water, and sediment. ANOSIM results showed significant differences (R = 0.669, *p* = 0.0001) among these three groups (Supplementary Figure S3), which implied the clustering was reasonable and reliable (Shang et al., 2023). Based on this result, the composition and structure of the microbial community were analyzed (Figure 2B). Their values are shown in Supplementary Table S2.

At the phylum level, there were significant differences in the relative abundance of the dominant microorganisms in the river, lake, and sediment (*p* < 0.05). The relative abundance of *Proteobacteria* (47.9 ± 5.4%, mean ± SD; 40.9 ± 5.1%), *Actinobacteriota* (16.6 ± 3.3%; 20.2 ± 3.3%), and *Firmicutes* (8.3 ± 3.3%; 18.2 ± 3.3%) in rivers and lakes was significantly higher than that in sediments (*p* < 0.05), with percentages of 1.5 and 1.3, 3.6 and 4.4, and 1.5 and 3.3 times those in the sediment, respectively. In the sediment, the relative abundances of *Chloroflexi* (12.0 ± 3.3%), *Desulfobacterota* (6.3 ± 0.8%), and *Acidobacteriota* (7.1 ± 2.3%) were significantly higher than those in rivers and lakes (*p* < 0.05), with abundances of 9.7 and 11.2%, 5.3 and 5.9%, and 4.5 and 6.7%, respectively.

At the genus level, *hgcI_clade, Pseudomonas, Comamonadaceae, Acinetobacter, Hydrogenophaga*, and *Exiguobacterium* had a higher relative abundance in river water and lake water, but their abundances in the sediment were <1%. However, *Thiobacillus, Anaerolineaceae*, and *Steroidobacteraceae* had a high abundance (>2.0%) in the sediment but were rarely detected in the river and lake. The abundance of *Arenimonas* was significantly higher in the lake water (5.3 ± 1.3%) (*p* < 0.05), 4.7 and 5.0% more than in the river water and sediment, respectively. The *Pseudomonas* abundance was significantly greater in the lake water (4.7 ± 1.3%), followed by the river water (2.2 ± 0.6%) and sediment (0.5 ± 0.1%) (*p* < 0.05). The relative abundance of *Comamonadaceae* in the river water (4.7 ± 2.2%) was slightly higher than in the other zones (*p* < 0.05). The *Acinetobacter* relative abundance was 9.3 ± 2.7% in the lake water, which was 5 and 9.3% higher than in the river water and the sediment zone, respectively (*p* < 0.05). In addition, the relative abundance of *Exiguobacterium* in the lake-water zone reached 12.1 ± 3.6%, which was significantly higher than in the river water (3.9 ± 0.8%) and sediment (0.1 ± 0.0%) (*p* < 0.05).

**Figure 3:**
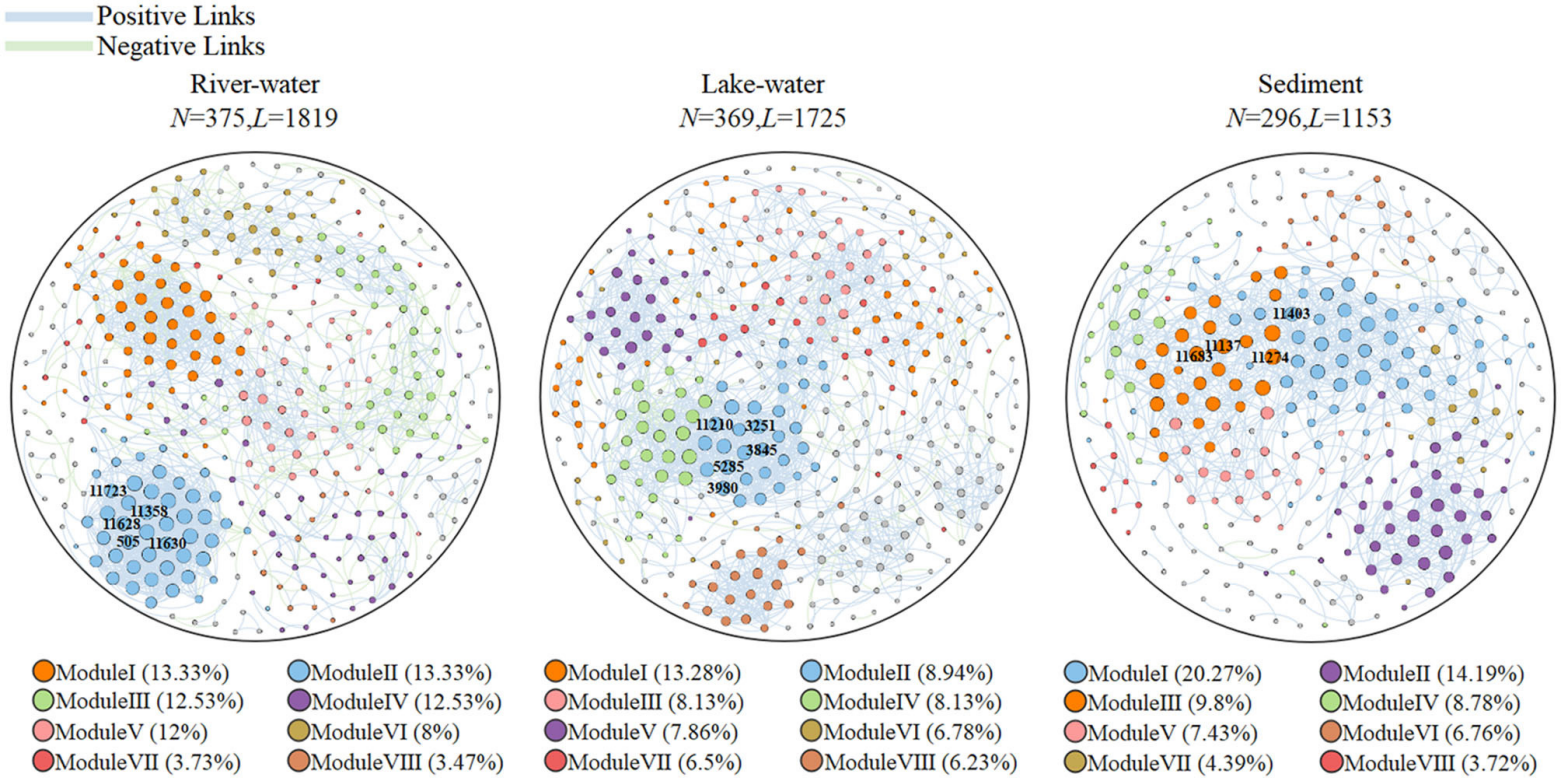
Topological networks of the river water, lake water, and sediment. *N* is the number of network nodes; *L* is the number of network edges. The blue edges represent positive connections, and the green edges represent negative connections. Modules I to VIII represent different aggregation modules. The microorganisms labeled with operational taxonomy unit (OTU) numbers are the keystone taxa.

Table 2 summarizes the characteristics of microbial molecular ecological networks. The microbial molecular ecological network constructed by our research was scientific and reasonable (see Supplementary Text description of the topological characteristics of the molecular ecological network). All ecological networks were dominated by positive correlations, with values ranging from 75.4 to 83.2% (Figure 3; Supplementary Table S3). It is worth noting that there was a significant difference among the results of average path length (*p* < 0.05), and the order was as follows: lake water (8.392) > river water (6.432) > sediment (6.077). The modularity of microbial communities showed significant differences (*p* < 0.05), and the order was lake water (0.728) > river water (0.726) > sediment (0.635). The keystone taxa can be used as indicators of the microbial community (Banerjee et al., 2018), and the keystone taxa were identified in this study (Supplementary Table S4; Figure 3).

**Table 2:** Topological properties of the empirical networks and their associated random networks in the different zones: River-water, Lake-water and Sediment zones.

		**Empirical network**							**Random network**		
**Zones**	**Nodes**	**Links**	**Average connective degree**	**Average path length**	**Average clustering coefficient**	**Modularity**	***R* ^2^**	**Small-world coefficient**	**Average path length (SD)**	**Average clustering coefficient (SD)**	**Modularity (SD)**
River-water	375	1,819	9.701	6.432^*^	0.584^**^	0.726^*^	0.791	3.384	2.944 (0.025)	0.079 (0.006)	0.259 (0.004)
Lake-water	369	1,725	9.350	8.392^*^	0.585^**^	0.728^*^	0.663	46.644	5.353 (0.067)	0.008 (0.004)	0.626 (0.007)
Sediment	296	1,153	7.791	6.077^*^	0.528^**^	0.635^*^	0.922	5.352	3.080 (0.032)	0.050 (0.005)	0.303 (0.005)

Values are means, with the SD in brackets. ^*^p < 0.05, ^**^p < 0.01.

### 3.3. Nitrogen and phosphorus metabolic pathways driven by key microbial communities and genes

**Figure 4:**
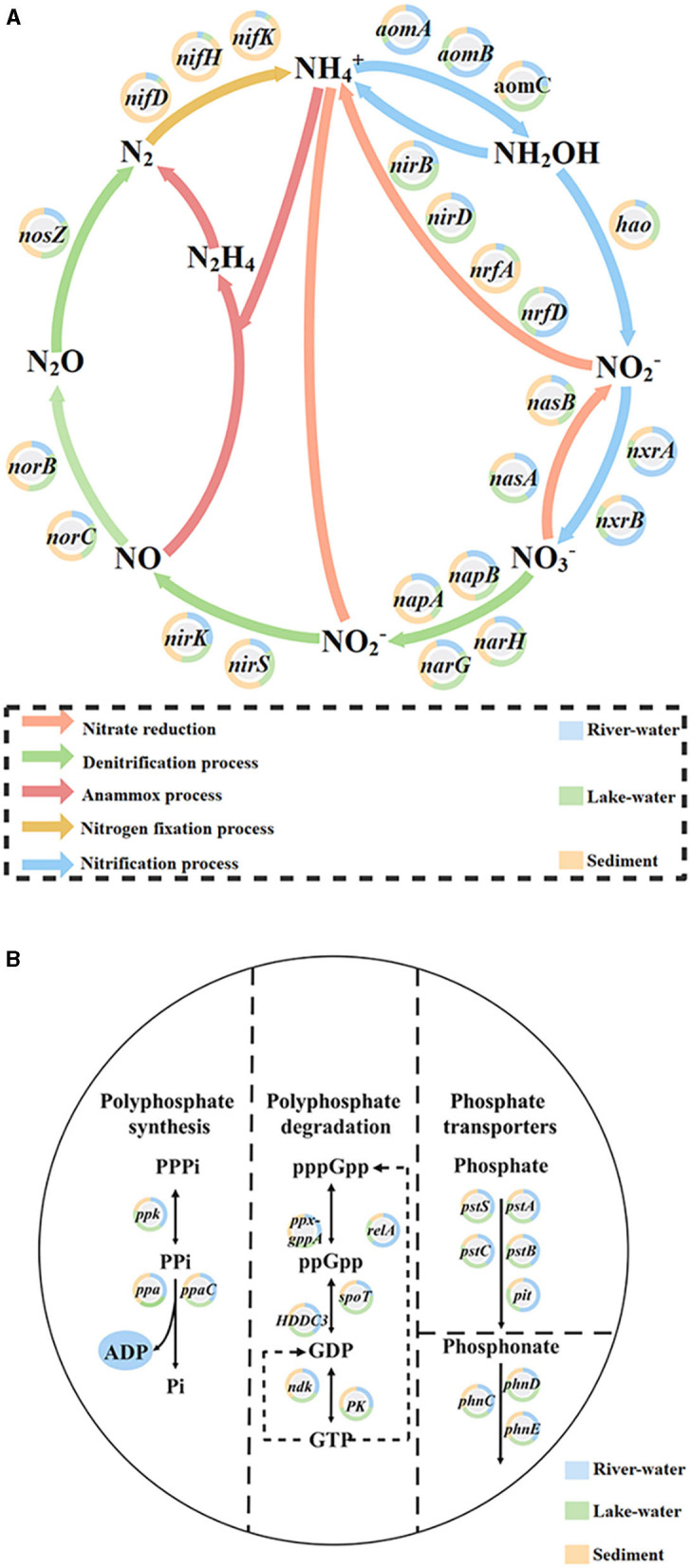
The proportion of **(A)** nitrogen and **(B)** phosphorus cycling-related genes expressed in river water, lake water, and sediment in each pathway.

The specific functions of microbial communities (such as nitrogen and phosphorus conversion) were closely related to metabolic pathways (Xiong et al., 2023). The results showed that the KO pathway (Supplementary Figures S4, S5) involved in the nitrogen and phosphorus cycle was highly expressed in lake water. Microbial functional genes were crucial for the transformation of nutrients (Li et al., 2022) to identify the genes that play key roles in the pathway of this river–lake system, we analyzed the expression levels of genes related to nitrogen and phosphorus pathways (Supplementary Figure S6) and the contribution of key genes (Figure 4). The results showed that the *nirB* (3.6%), *narG* (5.5%), narH (2.5%), and *nasA* (2.8%) genes related to the nitrate cycle and the *ppk* (6.5%), *pstA* (4.3%), *pstB* (4.2%), *pstC* (4.4%), and *pstS* (6.4%) genes related to the phosphate cycle were highly expressed in all samples (Supplementary Figure S6), and most (nitrate: *nirB*/*narG*/*narH/nasA*; phosphorus: *pstA*/*pstB*/*pstC*/*pstS*) were expressed significantly higher in the lake water than in the river water and sediment (Figure 4). These genes are closely related to nitrate reduction, phosphate absorption, and transport (Yu et al., 2018; Li et al., 2023; Liu et al., 2023). On this basis, the main microorganisms contributing these genes with high expression in lakes were identified in this study through the combination of species annotation and gene abundance calculation (Supplementary Figure S7; the detailed descriptions are shown in the Supplementary Text description of the analysis of key microorganisms contributing nitrogen and phosphorus genes). The results showed that *Pseudomonas, Acinetobacter, Nocardioides, Microbacterium, Agromyces*, and *Aeromonas* had a relatively high contribution of genes involved in the nitrate-phosphate cycle, especially in lake water. These microorganisms were defined as the key microorganisms that we should focus on in our study.

### 3.4. The influencing factors of nitrate and phosphate concentration

**Figure 5:**
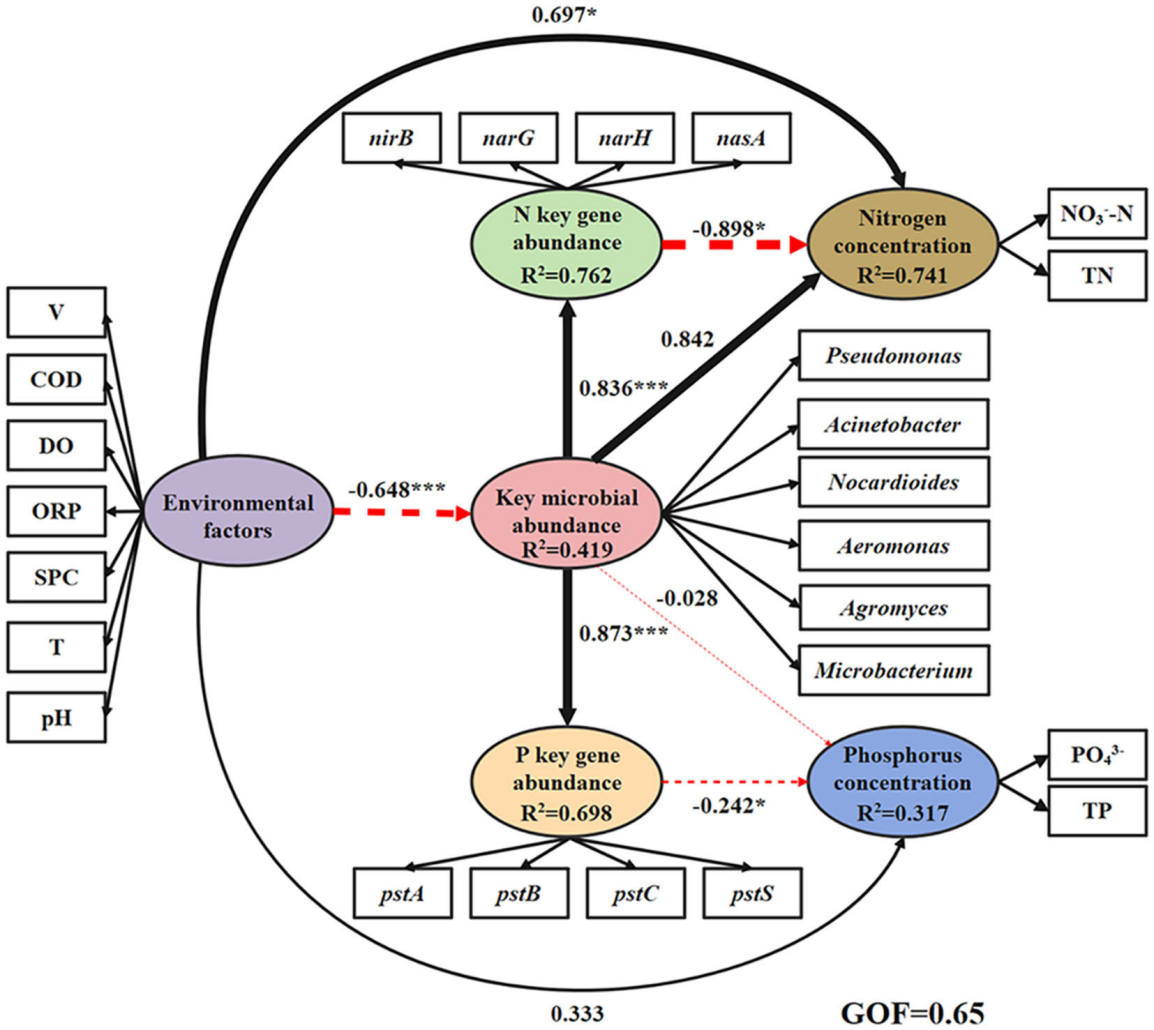
The relationship and influence of environmental factors, key microbial abundance, nitrogen/phosphorus cycling gene abundance, and nitrogen/phosphorus concentrations are shown by partial least squares path modeling (PLS-PM) in the water. GOF, Goodness-of-Fit (0.67 indicates a good fit). The black line represents the positive correlation, the red line represents the negative correlation, and the thickness of the line represents the size of the path coefficient (value range 0–1). *p < 0.05, ***p < 0.001.

The results showed that there was a significant positive correlation between the abundance of key microbial abundance and the genes promoting nitrogen reduction (0.836, *p* < 0.001) and phosphorus absorption and transport (0.873, *p* < 0.001, Figure 5). The effect of key gene abundance on nitrogen (−0.898, *p* < 0.001) and phosphorus (−0.242, *p* < 0.001) concentration showed a highly significant negative trend. The environmental factors showed strong positive effects on the concentrations of nitrogen (0.697) and phosphorus (0.333), and the effect on nitrogen was significant (*p* < 0.05). There was a significant negative effect (−0.648, *p* < 0.001) of environmental factors on the abundance of key microbial species. In the environmental factors, the factor load of V (0.727) and COD concentration (0.839) was obviously high and significant (*p* < 0.001), and showed strong significant positive correlations with the concentrations of nitrate and phosphate and negative correlations with key microbial abundance (Supplementary Table S5), respectively.

## 4. Discussion

This study revealed the key rate-limiting steps of microbial-mediated nitrogen and phosphorus cycling in a highly regulated river–lake system. Our original goals were confirmed. Specifically, we found that the concentration of nitrogen and phosphorus in lake water was significantly lower than that in river water; that is, the water quality was more stable. In addition, the stability of the microbial community and the abundance of key microorganisms associated with nitrogen and phosphorus conversion were higher in lake water. Key microorganisms and genes-driven denitrification and phosphorus absorption/transport processes become key biochemical rate-limiting steps. COD concentration and flow velocity were the most important environmental variables that affect key microorganisms and genes.

### 4.1. Effects of physicochemical characteristics on microbial communities in different zones

Our study has revealed that flow velocity and COD concentration in river water were obviously higher than in lake water. In the Dongping river–lake system, the water surface in the lake zone was wide and the flow velocity was relatively slow (Liu et al., 2022), while in the inflow area, the water surface was narrow and the flow velocity was fast (Liu et al., 2016). During the regulation period, there was often a huge water impact, which undoubtedly increased the flow velocity in the outflow area (Zhang et al., 2012). The Dawen River mainly passes through rural areas, and organic matter in soil, farm compost, and decaying plants will enter the river with rainwater and irrigation water, resulting in a high COD concentration (Lv et al., 2018). In addition, some of the pollutants generated by human activities were discharged into the river and accumulated at the gates. When the flood was discharged, the gates were opened, and the pollutants were released along with the flow (Piirsoo et al., 2018; Watkins et al., 2019). This may also lead to higher COD concentration in the outflow zones.

We found that high flow velocity in river water zones has negative effects on the stability of microbial communities. In lake water zones with relatively low flow velocity, the microbial network has the longest path length, which suggests that when the environment is disturbed (e.g., through water inflows or releases), the response rate is the slowest for microorganisms in the lake zone, and the impact of environmental disturbance will not spread quickly to the whole microbial molecular ecological network (Jeanbille et al., 2016). The modularity of the microbial community in the lake water was the highest, which was similar to the study of Khu et al. (2023), who found that the low flow velocity conditions were more conducive to microbial growth and formed a higher degree of modularity. The high degree of modularity indicated that the function of the microbial community was more complex, and the microbial community has a strong ability to resist environmental stress (Feng et al., 2017). In addition, the numerous positive correlations in lake water represented that microorganisms were more inclined toward cooperative relationships, which promoted the stability of microbial communities (Layeghifard et al., 2017). The influence of higher flow velocity and COD concentration on the abundance of key microorganisms was negative in our study. When the water flow velocity increased, the desorption force generated by flow shear reduced the adsorption of microorganisms to suspended matter in the water, inhibiting the formation of biofilm (Tian et al., 2017). High COD concentration indicated that the water accepts more organic pollutants, and the biodegradation of these organic substances will consume DO (Liu et al., 2021). Most of the key microorganisms found in our study were aerobic denitrifying bacteria and phosphorus-accumulating organisms (PAOs), which need DO for their own reproduction (Zhao et al., 2022; Shen et al., 2023), so high COD concentration will inhibit the abundance of these key microorganisms.

### 4.2. The rate-limiting steps in microorganism-mediated nitrogen and phosphorus cycles

We found that total nitrogen and total phosphorus concentrations depend on ${\text{NO}}_{3}^{-}$-N and ${\text{PO}}_{4}^{3-}$ concentrations in the water column. Therefore, we conclude that controlling ${\text{NO}}_{3}^{-}$-N and ${\text{PO}}_{4}^{3-}$ concentrations was the key to maintaining water quality stability during water regulation. Previous studies have also confirmed our findings about nitrate nitrogen and phosphate, which are often used as key indicators in water quality monitoring to judge the deterioration of water quality for subsequent control (Rashid and Romshoo, 2013; Bhurtun et al., 2019). Hence, microorganism-mediated steps that can affect nitrate and phosphate conversion become the focus of this study, which may also be a key step in water quality stability during water regulation.

We found that *Pseudomonas, Acinetobacter*, and *Microbacterium* were dominant and keystone taxa in the lake-water (Figures 2, 3) zones. The dominant microorganisms with high biomass can affect some broad community processes, and rare keystone taxa are more visible in narrower processes; they are the drivers of microbial community structure and function, such as a particular one in the nitrogen and phosphorus cycles (Banerjee et al., 2018). In addition, we found that *Pseudomonas* mainly contributes *nirB, narG*, and *narH* genes, while *Acinetobacter* can contribute *nirB, pstB, pstC*, and *pstS* genes. *Microbacterium* contributed the *nasA, pstA*, and *pstB* genes. For the nitrogen cycle, *nirB* is the gene corresponding to the nitrite assimilation reductase, *narG*/*narH* corresponds to denitrification, and *nasA* corresponds to nitrate assimilation reduction (Wang et al., 2018). For the phosphorus cycle, *pstA, pstB, pstC*, and *pstS* were the corresponding genes for phosphate absorption and transport, and these were the key to the function of PAOs (Singleton et al., 2021). In addition, previous studies have confirmed our findings: *Pseudomoas* was confirmed to be aerobic denitrifying bacteria (Shen et al., 2023), and the presence of *Pseudomonas* and *Acinetobacter* can accelerate the rate of nitrate reduction in sewage (Hu et al., 2022; Xiao et al., 2023). *Pseudomonas* and *Acinetobacter* are typical PAOs, that can realize excessive phosphate absorption through the aerobic phosphorus absorption process (He et al., 2020). *Microbacterium* was found to be denitrifying bacteria (Tang et al., 2023) and have good phosphorus removal potential (Wang et al., 2016). Hence, these microorganisms contribute significantly to the lower nitrogen and phosphorus concentrations in the lake zone. Hence, these dominant and keystone taxa may dominate the denitrification, phosphate absorption, and transport pathways to become key rate-limiting steps for water quality stabilization in this river–lake system.

### 4.3. Analysis of key factors affecting nitrogen and phosphorus concentration and water quality improvement strategies

We found that the inhibition of key genes on nitrogen and phosphorus concentrations was significant. This also showed that the key genes defined in this study can indeed promote the processes of denitrification and phosphorus absorption and transport. In addition, the increase in the abundance of key microorganisms was significantly beneficial to the abundance of nitrogen and phosphorus genes. These further confirmed the importance of microorganism-mediated denitrification, phosphorus absorption, and transport pathways for water quality stability. However, we found that high flow velocity and COD concentration may not be conducive to the stability of water quality during the regulation period. Hydrological disturbance, especially the increase in flow velocity, will cause the exchange of sediments and water in shallow lakes (Tang X. et al., 2020; Luo et al., 2022), resulting in the release of nitrogen and phosphorus from the sediments, which further increases the concentration of pollutants in the water phase (Huang et al., 2015; Peng et al., 2021). The higher COD concentration may be unfavorable to the propagation of aerobic denitrifying microorganisms and PAOs (as mentioned in Section 4.1), resulting in higher nitrogen and phosphorus concentrations in water.

**Figure 6:**
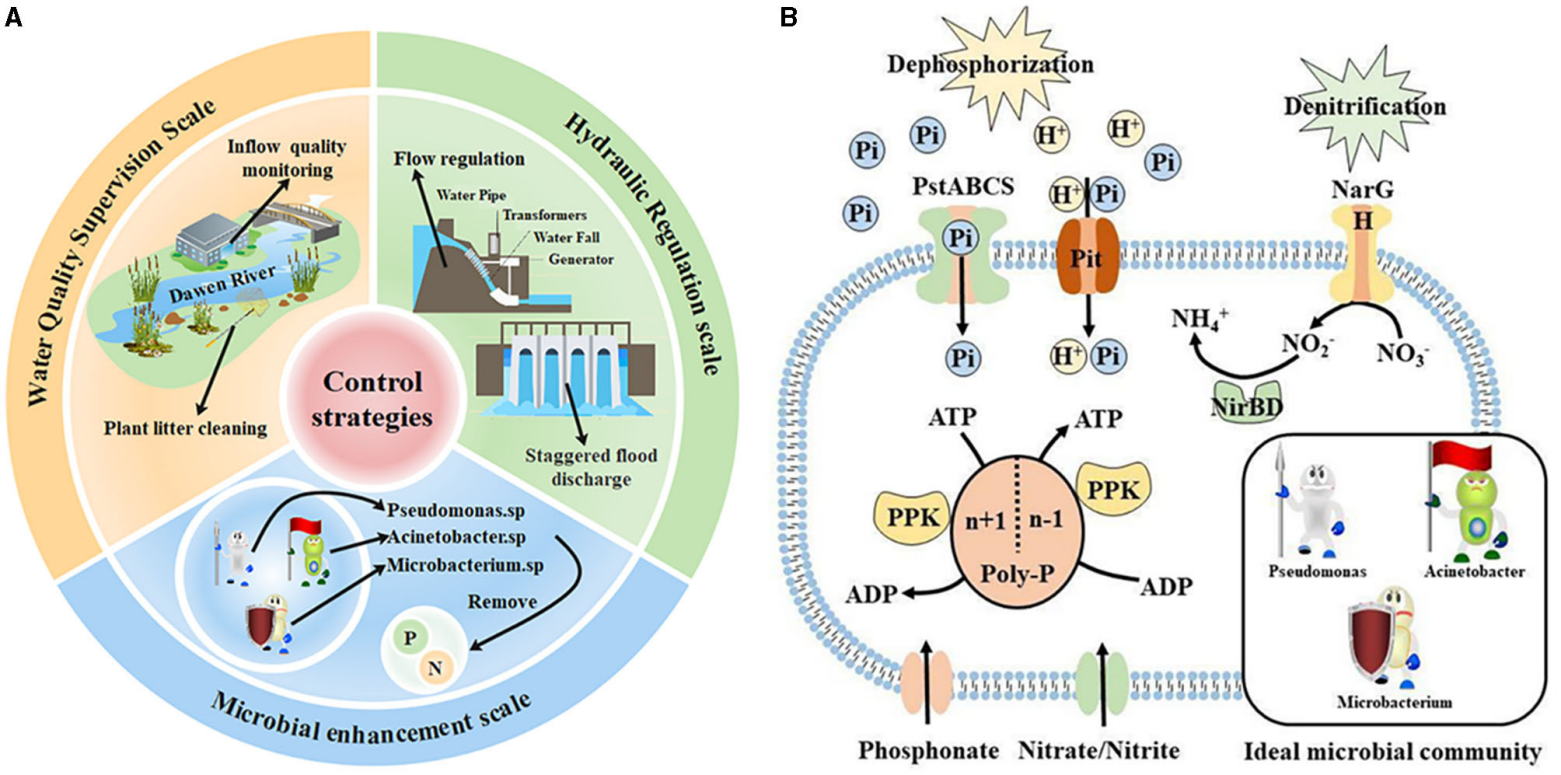
**(A)** Water quality stability improvement strategies and **(B)** ideal microbial community gene function model.

In summary, in this study, maintaining the abundance of key microorganisms (especially the dominant and keystone taxa) and controlling the regulation flow as well as the concentration of pollutants accepted by the water body were crucial to maintaining the stability of water quality during hydrological regulation. Based on this, important strategies were proposed to protect water quality in this study (Figure 6A). On the scale of hydrological regulation, we suggest adopting the mode of cross-peak flood regulation to reduce the kinetic energy of flow to a certain extent and weaken the exchange between water and sediment. In terms of the water quality supervision scale of the accepted water bodies, we suggest increasing the monitoring of the incoming water quality of the Dawen River, removing the sewage behavior along the river, and regularly cleaning up the plant spoilage litter in the river to reduce the organic matter content. On the scale of the microbial community, we propose to add key microbial complex agents containing *Pseudomonas, Acinetobacter*, and *Microbacterium* in the inflow and outflow zones during the regulation period. Through the above strategies, the ideal microbial community gene function model was finally achieved (Figure 6B). It can promote nitrogen and phosphorus removal to maintain water quality stability.

## 5. Conclusion

In this study, we have revealed the important influence of microorganisms on water quality stability in a highly regulated river–lake system using the Dongping river–lake system as the case study. The lake zones showed better water quality compared with the discharge and inflow zones. In sediments, there was no significant difference in nitrogen and phosphorus concentrations. Denitrification and phosphorus absorption and transport mediated by key microorganisms were key rate-limiting steps for water quality stability. *Pseudomonas, Acinetobacter*, and *Microbacterium* have the most important effects on denitrification, phosphorus absorption, and transport steps. The increase in flow velocity and nutrient load was unfavorable to key microbial abundance. This study will help us ensure the quality and health of water during the process of water transfers and flow regulation engineering.

## Data availability statement

The original microbial sequencing data presented in the study are publicly available. This data has been uploaded to the National Center for Biotechnology Information (NCBI) database under the accession number PRJNA1009027 (https://www.ncbi.nlm.nih.gov/bioproject/PRJNA1009027). The original contributions presented in the study are included in the article/Supplementary material. Further inquiries can be directed to the corresponding authors.

## Author contributions

JD: Data curation, Methodology, Writing—original draft. WY: Funding acquisition, Supervision, Writing—review and editing. XL: Data curation, Investigation, Writing—review and editing. QZ: Data curation, Writing—review and editing. WD: Data curation, Writing—review and editing. CZ: Data curation, Writing—review and editing. HL: Writing—review and editing. YZ: Writing—review and editing.
