# Supplementary material for: Unraveling the rate-limiting step in microorganisms' mediation of denitrification and phosphorus absorption/transport processes in a highly regulated river-lake system
Source: Front Microbiol. 2023 Oct 13;14:1258659. doi: 10.3389/fmicb.2023.1258659 (PMC10613053; doi:10.3389/fmicb.2023.1258659)
Supplement: Supplementary file 1 [file Data_Sheet_1.docx]

**Supplementary Material**

# Unraveling the rate-limiting step in microorganisms’ mediate denitrification and phosphorus absorption/transport processes in a highly regulated river–lake system

Jiewei DING^1^, Wei YANG^1*^, Xinyu LIU^1^, Qingqing ZHAO^2^, Weiping DONG^1^, Chuqi ZHANG^1^, Haifei LIU^1^, Yanwei ZHAO^1^

**Affiliation:**

1. State Key Laboratory of Water Environment Simulation, School of Environment, Beijing Normal University, Beijing 100875, China

2. Shandong Provincial Key Laboratory of Applied Microbiology, Ecology Institute, Qilu University of Technology (Shandong Academy of Sciences), Ji’nan, 250103, China

Correspondence:

Prof. Wei Yang

Address: School of Environment, Beijing Normal University, Beijing 100875, China

E-mail address: yangwei@bnu.edu.cn

**Supplementary figures**


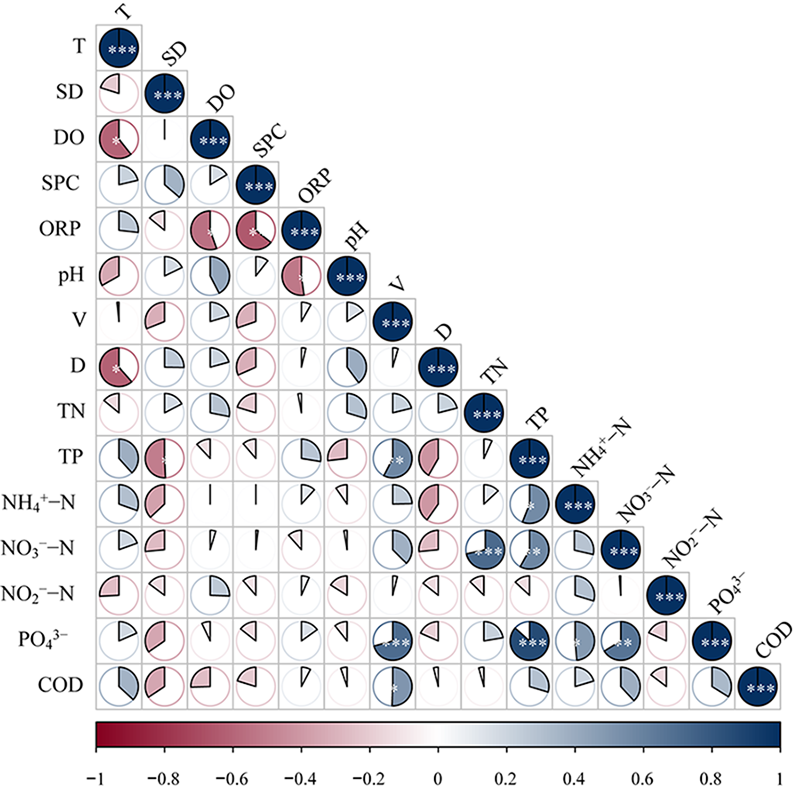


**Figure S1** Pearson correlation between physical and chemical properties. Red to blue indicates a negative correlation to a positive correlation, and the larger the sector, the stronger the correlation. **p* < 0.05, ***p* < 0.01, ****p* < 0.001.


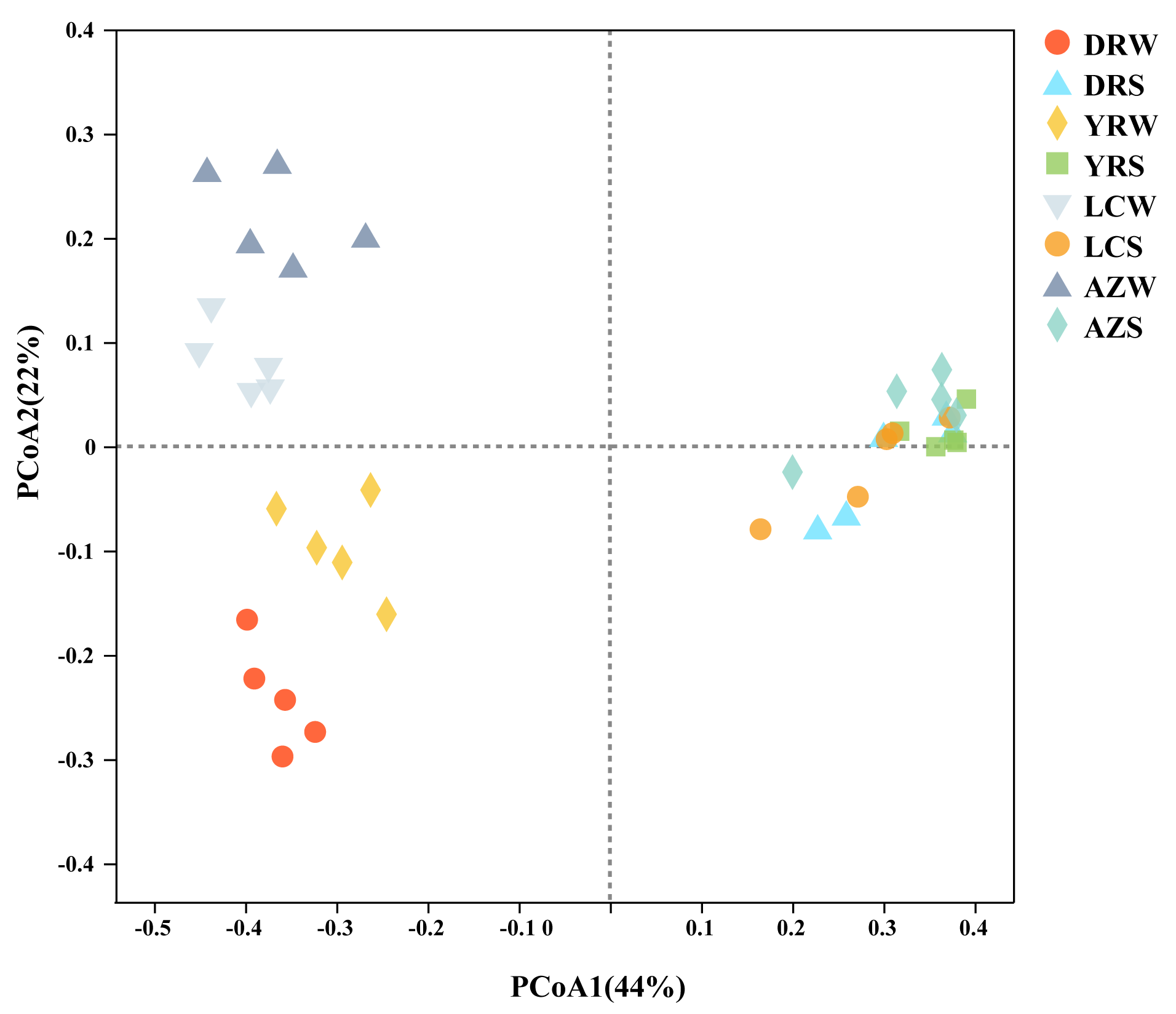


**Figure S2** Principal coordinates analysis (PCoA) of microbial communities. DR, Dawen river inflow zone; LC, lake center zone; AZ, aquaculture zone; YR, Yellow River outflow zone. W and S stand for water column and sediment, respectively.


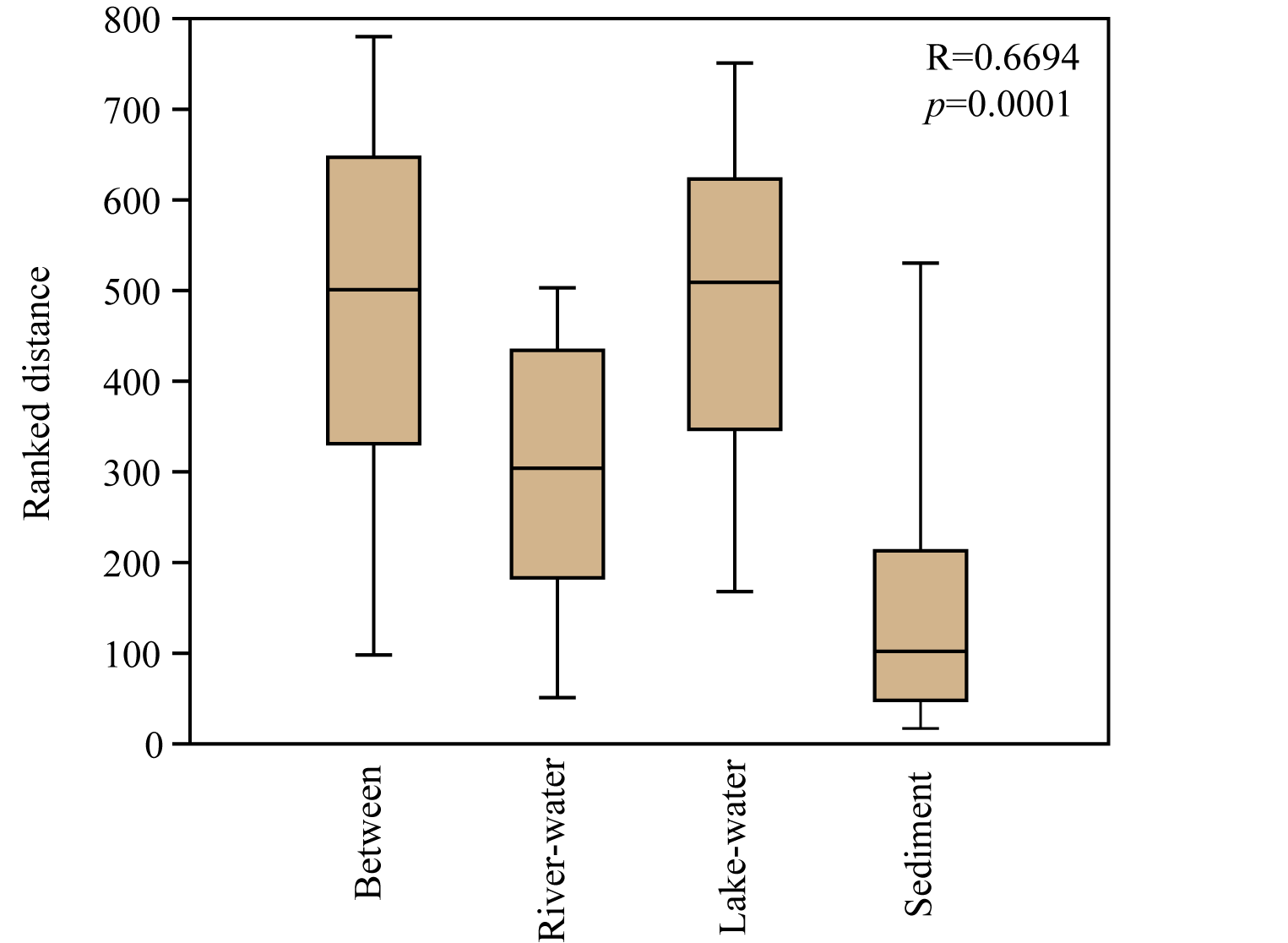


**Figure S3** Analysis of similarities (ANOSIM) reflecting the differences between groups clustering algorithm.


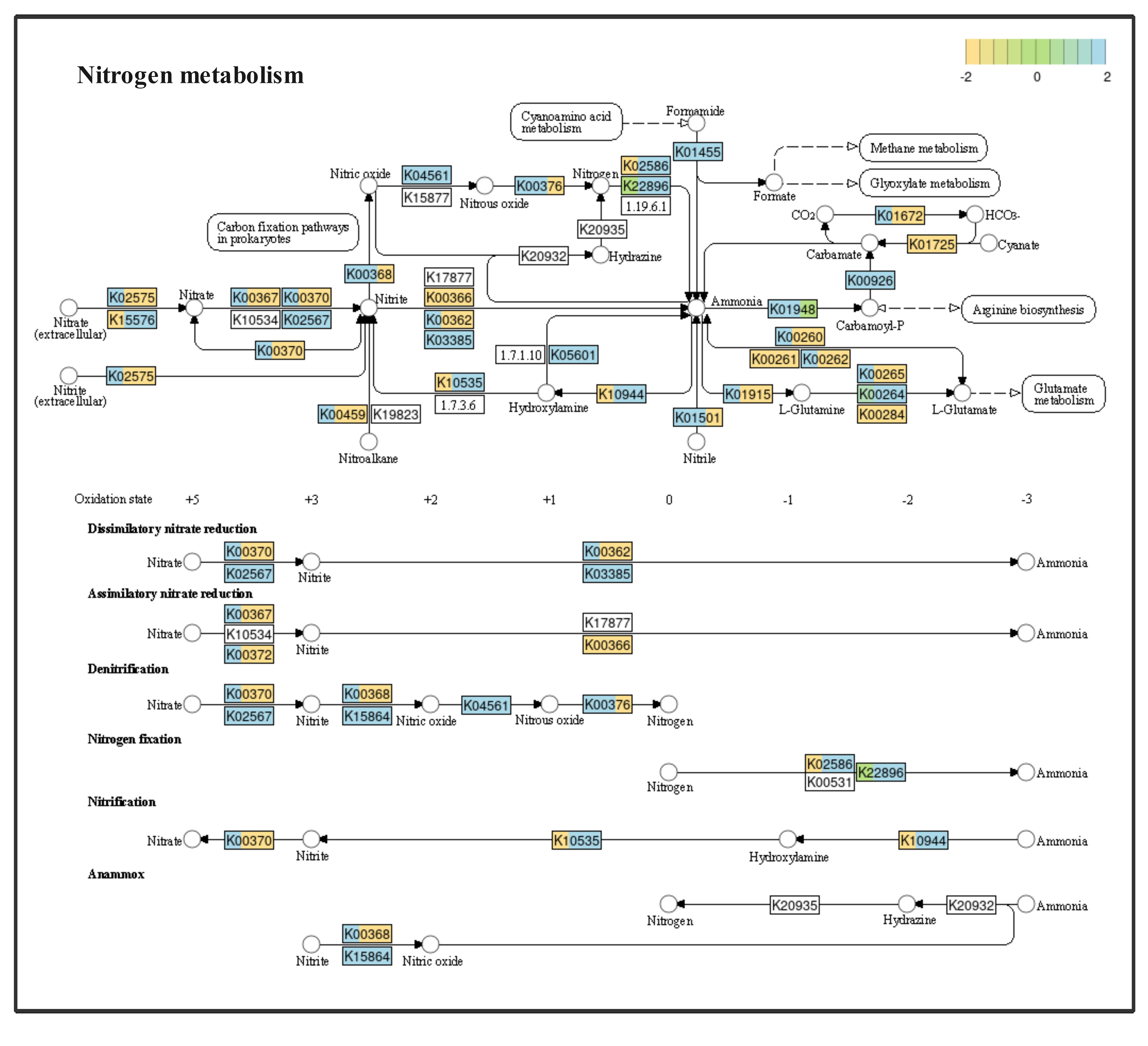


**Figure S4** KEGG pathway analysis of nitrogen metabolism. Each channel color was divided from left to right into three sections, namely, lake-water/river-water expression difference, sediment/river-water expression difference, and sediment/lake-water expression difference.


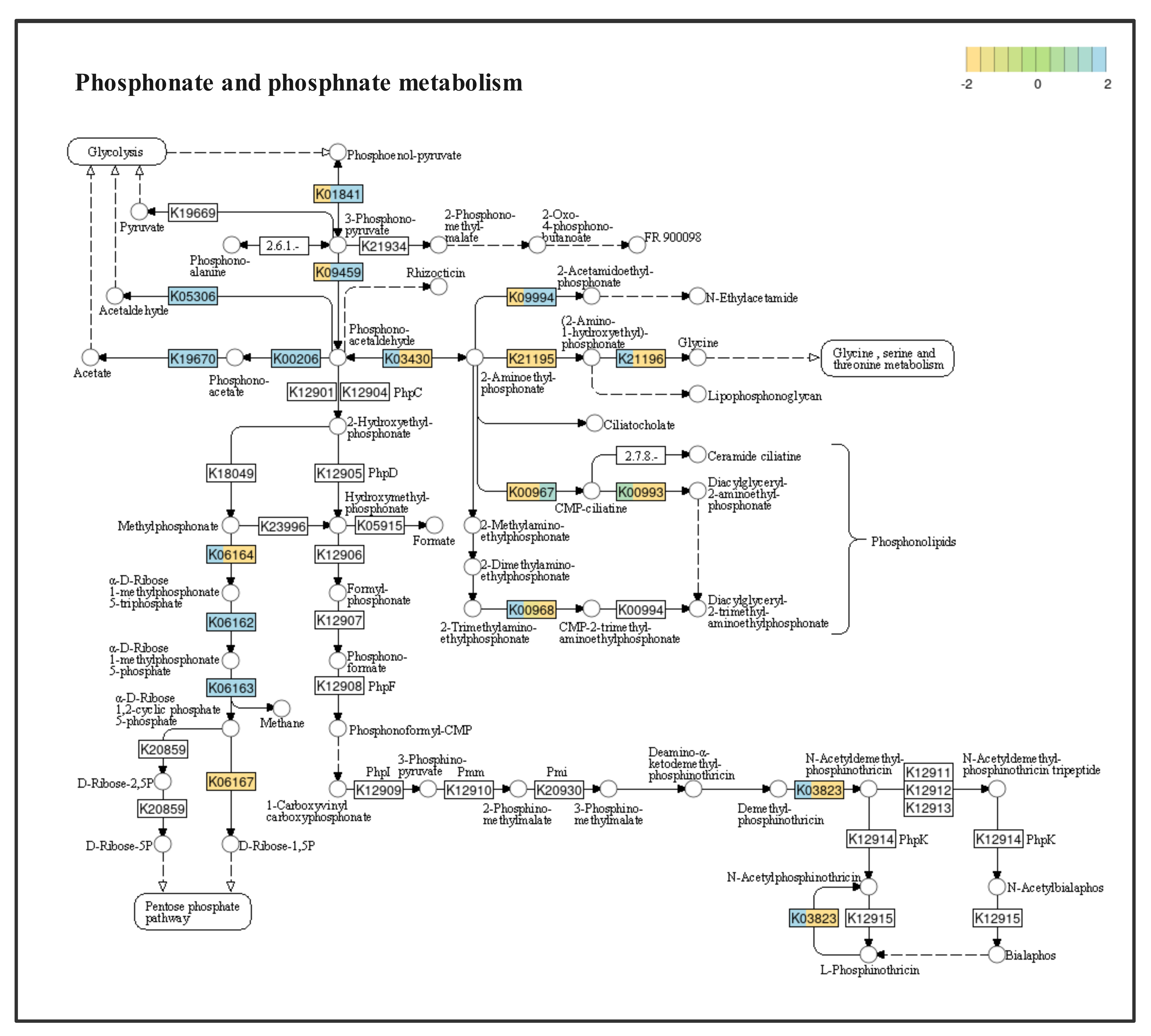
**Figure S5** KEGG pathway analysis of phosphonate and phosphnate metabolism. Each channel color was divided from left to right into three sections, namely, lake-water/river-water expression difference, sediment/river-water expression difference, and sediment/lake-water expression difference.


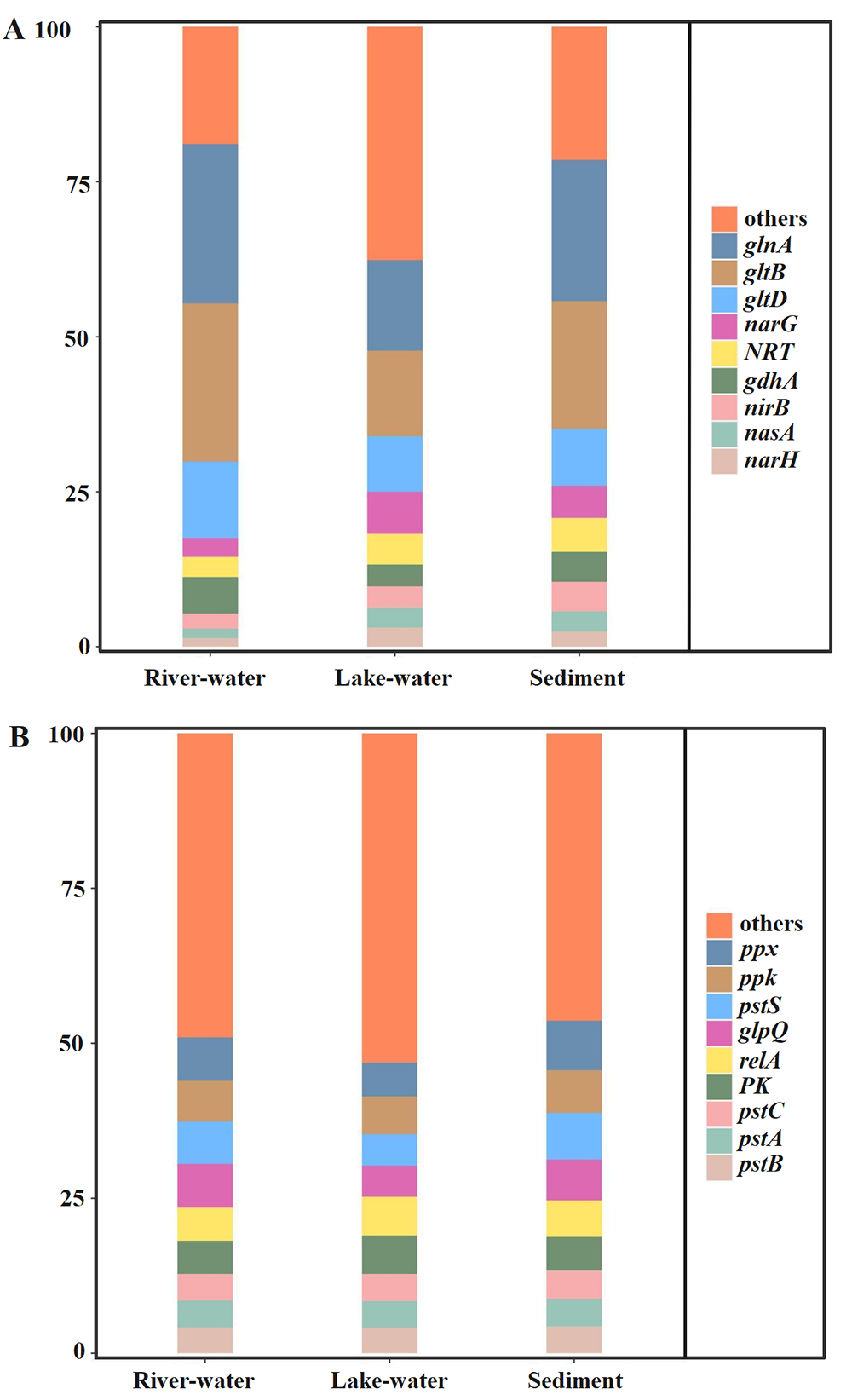


**Figure S6** High expression genes associated with (A) nitrogen and (B) phosphorus cycling in river-water, lake-water and sediment.


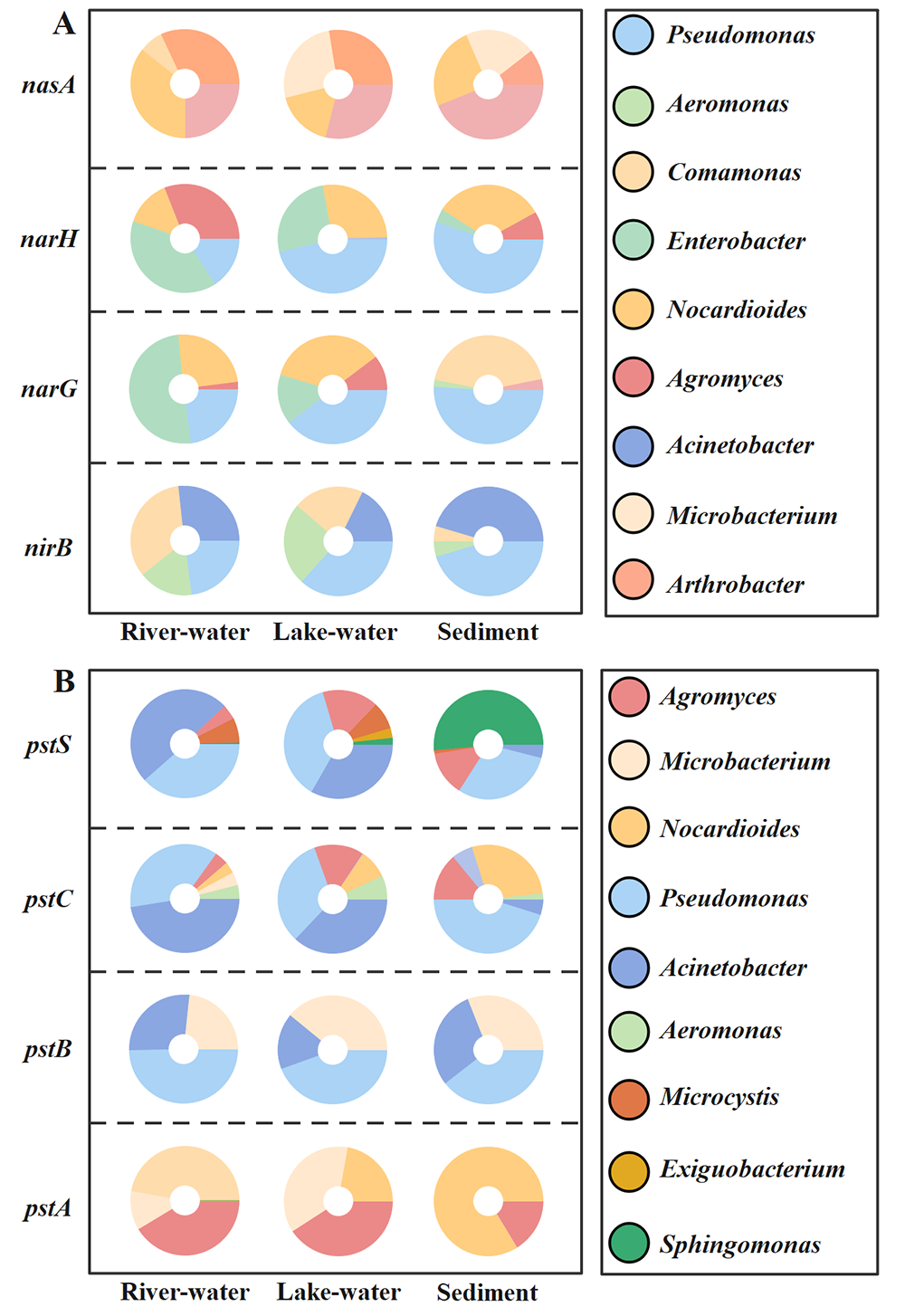


**Figure S7** Contribution of major microorganisms to high abundance of (A) nitrogen (*nirB*/*narG*/*narH*/*nasA*) and (B) phosphorus (*pstA*/*pstB*/*pstC*/*pstS*) cycle genes.

**Supplementary tables**

**Table S1** Test standards for the physical and chemical indexes. Abbreviations: COD, chemical oxygen demand; NH_4_^+^-N, ammonia nitrogen; NO_3_^-^-N, nitrate nitrogen; NO_2_^-^-N, nitrite nitrogen; PO_4_^3-^ , phosphate TN, total nitrogen; TP, total phosphorus.

| Samples | Indexes | Testing standard from China’s Ministry of Ecology and Environment |
| --- | --- | --- |
| Water | COD (mg/L) | HJ 828-2017 |
|  | TN (mg/L) | HJ 636-2012 |
|  | NH_4_^+^-N (mg/L) | HJ 535-2009 |
|  | NO_3_^-^-N (mg/L) | HJ 84-2016 |
|  | NO_2_^-^-N (mg/L) | HJ 84-2016 |
|  | TP (mg/L) | GB/T11893-1989 |
|  | PO_4_^3-^ (mg/L) | HJ 84-2016 |
| Sediment | COD (mg/g) | Patent: CN106153861A,2016-11-23 |
|  | TN (mg/g) | HJ 717-2014 |
|  | NH_4_^+^-N (mg/g) | HJ 634-2012 |
|  | NO_3_^-^-N (mg/g) | HJ 634-2012 |
|  | NO_2_^-^-N (mg/g) | HJ 634-2012 |
|  | TP (mg/g) | HJ 632-2011 |
|  | PO_4_^3-^ (mg/g) | NY/T 1121.7-2014 |

**Table S2** The structure and composition of the microorganism communities at the phylum and genus levels. Values are mean ± SD. Genus that could not be defined were traced back to the family and order level. For the taxa, o represents an order, f represents a family, and g represents a genus.

| Levels | Microorganism | Samples | |
| --- | --- | --- | --- |
| Phylum | *Proteobacteria* | River-water | 47.9±5.4a |
|  |  | Lake-water | 40.85±5.1a |
|  |  | Sediment | 31.675±3.3b |
|  | *Actinobacteriota* | River-water | 16.6±2.0a |
|  |  | Lake-water | 20.2±1.2a |
|  |  | Sediment | 4.6±0.8b |
|  | *Bacteroidota* | River-water | 8.2±2.6a |
|  |  | Lake-water | 5.7±1.6a |
|  |  | Sediment | 4.85±1.8b |
|  | *Cyanobacteria* | River-water | 5.85±1.6a |
|  |  | Lake-water | 8.6±2.3a |
|  |  | Sediment | 0.775±0.1b |
|  | *Firmicutes* | River-water | 8.3±1.9b |
|  |  | Lake-water | 18.2±5.4a |
|  |  | Sediment | 5.6±2.4c |
|  | *Planctomycetota* | River-water | 2.8±1.2a |
|  |  | Lake-water | 2.2±0.8a |
|  |  | Sediment | 3.7±1.2a |
|  | *Verrucomicrobiota* | River-water | 1.7±0.6b |
|  |  | Lake-water | 1.5±0.2b |
|  |  | Sediment | 2.6±0.8a |
|  | *Chloroflexi* | River-water | 2.3±1.0b |
|  |  | Lake-water | 0.8±0.2b |
|  |  | Sediment | 12.0±3.6a |
|  | *Gemmatimonadota* | River-water | 0.6±0.2b |
|  |  | Lake-water | 0.3±0.1b |
|  |  | Sediment | 1.3±0.2a |
|  | *Acidobacteriota* | River-water | 1.6±0.3b |
|  |  | Lake-water | 0.4±0.1c |
|  |  | Sediment | 7.1±2.3a |
|  | *unclassified* | River-water | 0.4±0.1b |
|  |  | Lake-water | 0.3±0.1b |
|  |  | Sediment | 1.2±0.0a |
|  | *Myxococcota* | River-water | 0.5±0.2a |
|  |  | Lake-water | 0.3±0.0a |
|  |  | Sediment | 2.1±0.3a |
|  | *Desulfobacterota* | River-water | 1.0±0.0b |
|  |  | Lake-water | 0.4±0.0b |
|  |  | Sediment | 6.3±2.1a |
|  | *Nitrospirota* | River-water | 0.3±0.0a |
|  |  | Lake-water | 0.1±0.0a |
|  |  | Sediment | 2.2±0.6a |
|  | *Campilobacterota* | River-water | 0.0±0.0b |
|  |  | Lake-water | 0.0±0.0b |
|  |  | Sediment | 3.0±0.7a |
| Genus | *Arenimonas* | River-water | 0.6±0.0b |
|  |  | Lake-water | 5.3±1.3a |
|  |  | Sediment | 0.3±0.0b |
|  | *hgcI_clade* | River-water | 4.4±1.3a |
|  |  | Lake-water | 8.9±3.3a |
|  |  | Sediment | 0.0±0.0b |
|  | *Pseudomonas* | River-water | 2.2±0.6a |
|  |  | Lake-water | 4.7±1.3a |
|  |  | Sediment | 0.5±0.1b |
|  | *unclassified_f_Comamonadaceae* | River-water | 4.7±2.2a |
|  |  | Lake-water | 3.2±1.2a |
|  |  | Sediment | 2.2±1.0ba |
|  | *norank_f_norank_o_Chloroplast* | River-water | 3.7±1.3a |
|  |  | Lake-water | 4.2±1.6a |
|  |  | Sediment | 0.35±3.3b |
|  | *Arthrobacter* | River-water | 3.9±1.0a |
|  |  | Lake-water | 1.0±0.2b |
|  |  | Sediment | 0.1±0.0b |
|  | *Acinetobacter* | River-water | 4.3±1.8b |
|  |  | Lake-water | 9.3±2.7a |
|  |  | Sediment | 0.0±0.0c |
|  | *Hydrogenophaga* | River-water | 2.8±0.8a |
|  |  | Lake-water | 2.1±1.3a |
|  |  | Sediment | 0.5±0.0b |
|  | *Exiguobacterium* | River-water | 3.9±0.8b |
|  |  | Lake-water | 12.1±3.6a |
|  |  | Sediment | 0.1±0.0c |
|  | *Planococcus* | River-water | 1.8±0.6b |
|  |  | Lake-water | 3.0±1.4a |
|  |  | Sediment | 1.0±0.4b |
|  | *Cyanobium_PCC-*6307 | River-water | 1.6±0.7b |
|  |  | Lake-water | 3.5±1.5a |
|  |  | Sediment | 0.2±0.0c |
|  | *norank_f_Steroidobacteraceae* | River-water | 1.9±0.8a |
|  |  | Lake-water | 0.4±0.1b |
|  |  | Sediment | 2.0±0.6a |
|  | *norank_f_Anaerolineaceae* | River-water | 0.6±0.1b |
|  |  | Lake-water | 0.1±.0b |
|  |  | Sediment | 4.0±1.4a |
|  | *Thiobacillus* | River-water | 0.4±0.0b |
|  |  | Lake-water | 0.2±0.0b |
|  |  | Sediment | 4.4±1.7a |

**Table S3** The numbers and proportions of positive and negative correlations for the microbial molecular ecological networks in the three different groups of the river–lake system.

| Sample | Positive correlation | Proportion (%) | Negative correlation | Proportion (%) |
| --- | --- | --- | --- | --- |
| River-water | 1519 | 83.51 | 300 | 16.49 |
| Lake-water | 1633 | 94.67 | 92 | 5.33 |
| Sediment | 1150 | 99.74 | 3 | 0.26 |

**Table S4** The keystone ranking for the top 1% of the taxa in each topological network and the relevant taxonomic information. “Module” represents a group of taxa adapted to the same ecological niche and that tend to co-occur. OTU, operational taxonomic unit. For the taxa, o represents an order, f represents a family, and g represents a genus.

| Samples | OTU ID | Module | Phylum | Lowest taxonomic information | Degree |
| --- | --- | --- | --- | --- | --- |
| River-water | OTU11723 | Ⅱ | *Actinobacteriota* | *o__Micrococcales* | 39 |
|  | OTU11630 | Ⅱ | *Firmicutes* | *g__Planococcus* | 38 |
|  | OTU11628 | Ⅱ | *Proteobacteria* | *g__Devosia* | 38 |
|  | OTU11358 | II | *Bacteroidota* | *g__Cnuella* | 37 |
|  | OTU505 | Ⅱ | *Nitrospirota* | *g__ Nitrosomonas* | 37 |
| Lake-water | OTU3845 | Ⅱ | *Chloroflexi* | *f__Anaerolineaceae* | 25 |
|  | OTU3251 | Ⅱ | *Desulfobacterota* | *g__Geothermobacter* | 25 |
|  | OTU11210 | Ⅱ | *Actinobacteriota* | *g__Microbacterium* | 23 |
|  | OTU5285 | II | *Acidobacteriota* | *o__Aminicenantales* | 23 |
|  | OTU3980 | Ⅱ | *Bacteroidota* | *f__Bacteroidetes_vadinHA17* | 23 |
| Sediment | OTU11137 | Ⅲ | *Proteobacteria* | *f__Halieaceae* | 35 |
|  | OTU11274 | Ⅲ | *Proteobacteria* | *g__Denitratisoma* | 32 |
|  | OTU11403 | Ⅰ | *Bacteroidota* | *g__Fluviicola* | 32 |
|  | OTU11683 | Ⅲ | *Gemmatimonadota* | *f__Gemmatimonadaceae* | 32 |

**Table S5** Empirical correlation coefficient matrix of PLS model.

|  | V | COD | DO | NO^3-^-N | ORP | PO_4_^3-^ | SPC | T | pH | TN | TP | g__*Acinetobacter* | g__*Aeromonas* | g__*Agromyces* | g__*Microbacterium* | g__*Nocardioides* | g__*Pseudomonas* | narG | narH | nasA | nirB | pstA | pstB | pstC | pstS |
| --- | --- | --- | --- | --- | --- | --- | --- | --- | --- | --- | --- | --- | --- | --- | --- | --- | --- | --- | --- | --- | --- | --- | --- | --- | --- |
| V | 1.000 | 0.456 | 0.017 | 0.600 | 0.200 | 0.581 | -0.181 | 0.297 | 0.070 | 0.241 | 0.631 | -0.451 | -0.377 | -0.340 | -0.469 | -0.445 | -0.465 | -0.254 | -0.375 | -0.162 | -0.014 | -0.285 | 0.051 | -0.301 | -0.346 |
| COD | 0.456 | 1.000 | -0.221 | 0.756 | 0.137 | 0.342 | -0.166 | 0.427 | -0.086 | 0.832 | 0.365 | -0.598 | -0.489 | -0.493 | -0.569 | -0.518 | -0.578 | -0.691 | -0.750 | -0.742 | -0.354 | -0.487 | 0.087 | -0.417 | -0.513 |
| DO | 0.017 | -0.221 | 1.000 | -0.264 | -0.554 | -0.071 | 0.164 | -0.606 | 0.424 | -0.142 | -0.144 | 0.406 | -0.028 | 0.095 | 0.165 | 0.146 | 0.293 | 0.019 | 0.344 | 0.395 | -0.498 | 0.758 | 0.696 | 0.032 | 0.626 |
| NO_3_^-^-N | 0.600 | 0.756 | -0.264 | 1.000 | 0.306 | 0.205 | -0.080 | 0.439 | -0.128 | 0.571 | 0.255 | -0.440 | -0.343 | -0.361 | -0.403 | -0.383 | -0.423 | -0.435 | -0.542 | -0.467 | -0.127 | -0.368 | 0.038 | -0.318 | -0.342 |
| ORP | 0.200 | 0.137 | -0.554 | 0.306 | 1.000 | 0.151 | -0.643 | 0.267 | -0.521 | -0.100 | 0.141 | -0.421 | -0.009 | -0.112 | -0.253 | -0.205 | -0.309 | -0.001 | -0.283 | -0.351 | 0.345 | -0.588 | -0.571 | 0.053 | -0.516 |
| PO_4_^3-^ | 0.581 | 0.342 | -0.071 | 0.205 | 0.151 | 1.000 | -0.144 | 0.184 | -0.107 | 0.283 | 0.925 | -0.349 | -0.225 | -0.273 | -0.328 | -0.361 | -0.349 | -0.081 | -0.273 | -0.137 | 0.147 | -0.368 | -0.112 | -0.336 | -0.363 |
| SPC | -0.181 | -0.166 | 0.164 | -0.080 | -0.643 | -0.144 | 1.000 | 0.213 | 0.106 | 0.159 | -0.111 | 0.365 | 0.108 | 0.111 | 0.382 | 0.217 | 0.297 | 0.088 | 0.141 | 0.363 | 0.208 | 0.373 | 0.335 | -0.300 | 0.388 |
| T | 0.297 | 0.427 | -0.606 | 0.439 | 0.267 | 0.184 | 0.213 | 1.000 | -0.332 | 0.260 | 0.392 | -0.640 | -0.345 | -0.400 | -0.383 | -0.483 | -0.590 | -0.411 | -0.622 | -0.435 | 0.566 | -0.739 | -0.222 | -0.687 | -0.724 |
| pH | 0.070 | -0.086 | 0.424 | -0.128 | -0.521 | -0.107 | 0.106 | -0.332 | 1.000 | -0.075 | -0.218 | 0.252 | 0.008 | 0.131 | 0.099 | 0.180 | 0.172 | 0.006 | 0.181 | 0.272 | -0.298 | 0.417 | 0.419 | 0.065 | 0.369 |
| TN | 0.241 | 0.832 | -0.142 | 0.571 | -0.100 | 0.283 | 0.159 | 0.260 | -0.075 | 1.000 | 0.212 | -0.298 | -0.259 | -0.288 | -0.276 | -0.270 | -0.284 | -0.512 | -0.582 | -0.576 | -0.352 | -0.249 | 0.104 | -0.290 | -0.227 |
| TP | 0.631 | 0.365 | -0.144 | 0.255 | 0.141 | 0.925 | -0.111 | 0.392 | -0.218 | 0.212 | 1.000 | -0.530 | -0.418 | -0.451 | -0.501 | -0.549 | -0.542 | -0.243 | -0.407 | -0.243 | 0.197 | -0.494 | -0.045 | -0.527 | -0.522 |
| g__*Acinetobacter* | -0.451 | -0.598 | 0.406 | -0.440 | -0.421 | -0.349 | 0.365 | -0.640 | 0.252 | -0.298 | -0.530 | 1.000 | 0.800 | 0.817 | 0.924 | 0.900 | 0.983 | 0.786 | 0.894 | 0.845 | -0.061 | 0.851 | -0.021 | 0.642 | 0.909 |
| g__*Aeromonas* | -0.377 | -0.489 | -0.028 | -0.343 | -0.009 | -0.225 | 0.108 | -0.345 | 0.008 | -0.259 | -0.418 | 0.800 | 1.000 | 0.967 | 0.934 | 0.939 | 0.889 | 0.886 | 0.781 | 0.715 | 0.321 | 0.418 | -0.546 | 0.666 | 0.524 |
| g__*Agromyces* | -0.340 | -0.493 | 0.095 | -0.361 | -0.112 | -0.273 | 0.111 | -0.400 | 0.131 | -0.288 | -0.451 | 0.817 | 0.967 | 1.000 | 0.923 | 0.966 | 0.892 | 0.837 | 0.805 | 0.732 | 0.240 | 0.493 | -0.441 | 0.654 | 0.580 |
| g__*Microbacterium* | -0.469 | -0.569 | 0.165 | -0.403 | -0.253 | -0.328 | 0.382 | -0.383 | 0.099 | -0.276 | -0.501 | 0.924 | 0.934 | 0.923 | 1.000 | 0.955 | 0.963 | 0.839 | 0.830 | 0.816 | 0.245 | 0.634 | -0.279 | 0.566 | 0.721 |
| g__*Nocardioides* | -0.445 | -0.518 | 0.146 | -0.383 | -0.205 | -0.361 | 0.217 | -0.483 | 0.180 | -0.270 | -0.549 | 0.900 | 0.939 | 0.966 | 0.955 | 1.000 | 0.955 | 0.820 | 0.824 | 0.744 | 0.127 | 0.615 | -0.328 | 0.667 | 0.702 |
| g__*Pseudomonas* | -0.465 | -0.578 | 0.293 | -0.423 | -0.309 | -0.349 | 0.297 | -0.590 | 0.172 | -0.284 | -0.542 | 0.983 | 0.889 | 0.892 | 0.963 | 0.955 | 1.000 | 0.838 | 0.893 | 0.820 | 0.021 | 0.762 | -0.183 | 0.688 | 0.839 |
| narG | -0.254 | -0.691 | 0.019 | -0.435 | -0.001 | -0.081 | 0.088 | -0.411 | 0.006 | -0.512 | -0.243 | 0.786 | 0.886 | 0.837 | 0.839 | 0.820 | 0.838 | 1.000 | 0.900 | 0.850 | 0.379 | 0.446 | -0.520 | 0.696 | 0.550 |
| narH | -0.375 | -0.750 | 0.344 | -0.542 | -0.283 | -0.273 | 0.141 | -0.622 | 0.181 | -0.582 | -0.407 | 0.894 | 0.781 | 0.805 | 0.830 | 0.824 | 0.893 | 0.900 | 1.000 | 0.893 | 0.097 | 0.708 | -0.197 | 0.716 | 0.768 |
| nasA | -0.162 | -0.742 | 0.395 | -0.467 | -0.351 | -0.137 | 0.363 | -0.435 | 0.272 | -0.576 | -0.243 | 0.845 | 0.715 | 0.732 | 0.816 | 0.744 | 0.820 | 0.850 | 0.893 | 1.000 | 0.266 | 0.715 | -0.054 | 0.453 | 0.733 |
| nirB | -0.014 | -0.354 | -0.498 | -0.127 | 0.345 | 0.147 | 0.208 | 0.566 | -0.298 | -0.352 | 0.197 | -0.061 | 0.321 | 0.240 | 0.245 | 0.127 | 0.021 | 0.379 | 0.097 | 0.266 | 1.000 | -0.389 | -0.552 | -0.197 | -0.307 |
| pstA | -0.285 | -0.487 | 0.758 | -0.368 | -0.588 | -0.368 | 0.373 | -0.739 | 0.417 | -0.249 | -0.494 | 0.851 | 0.418 | 0.493 | 0.634 | 0.615 | 0.762 | 0.446 | 0.708 | 0.715 | -0.389 | 1.000 | 0.457 | 0.425 | 0.968 |
| pstB | 0.051 | 0.087 | 0.696 | 0.038 | -0.571 | -0.112 | 0.335 | -0.222 | 0.419 | 0.104 | -0.045 | -0.021 | -0.546 | -0.441 | -0.279 | -0.328 | -0.183 | -0.520 | -0.197 | -0.054 | -0.552 | 0.457 | 1.000 | -0.411 | 0.324 |
| pstC | -0.301 | -0.417 | 0.032 | -0.318 | 0.053 | -0.336 | -0.300 | -0.687 | 0.065 | -0.290 | -0.527 | 0.642 | 0.666 | 0.654 | 0.566 | 0.667 | 0.688 | 0.696 | 0.716 | 0.453 | -0.197 | 0.425 | -0.411 | 1.000 | 0.504 |
| pstS | -0.346 | -0.513 | 0.626 | -0.342 | -0.516 | -0.363 | 0.388 | -0.724 | 0.369 | -0.227 | -0.522 | 0.909 | 0.524 | 0.580 | 0.721 | 0.702 | 0.839 | 0.550 | 0.768 | 0.733 | -0.307 | 0.968 | 0.324 | 0.504 | 1.000 |

**Supplementary text**

**The small-world coefficient (σ) is calculated as follows**

| σ = γ / λ = (*C* / *C*_random_) / (*L* / *L*_random_) | (S1) |
| --- | --- |

where γ is the ratio of the clustering coefficient of an empirical network to that of a random network, λ is the ratio of the average path length of an empirical network to that of a random network, and *C* and *L* are the clustering coefficient and average path length in the empirical network, respectively; *C*_random_ and *L*_random_ are the clustering coefficient and average path length in the random network, respectively. If σ > 1, the empirical network usually has the characteristics of a small-world network.

**Microbial 16S rRNA and metagenomic sequencing**

We filtered the collected water samples through 0.22-μm polytef membrane filters to obtain samples from which we could extract DNA using the E.Z.N.A. soil DNA Kit (Omega Bio-tek, Norcross, GA, U.S.). After extraction, the DNA was detected using 1% agarose gel electrophoresis. Polymerase chain reaction amplification and Illumina sequencing were performed for the V4 region of the bacterial 16S rRNA gene using the primer pair 515F (5'-GTGYCAGCMGCCGCGGTAA-3') and 806R (5'-GGACTACNVGGGTWTCTAAT-3') primers. Purified amplicons were pooled in an equimolar and paired-end-sequenced on an Illumina MiSeq PE300 platform (Illumina, San Diego, CA, USA) according to the standard protocols by Majorbio Bio-Pharm Technology Co. Ltd. (Shanghai, China). We performed operational taxonomy unit (OTU) cluster and species classification analysis using version 7.0.1090 of Uparse (http://drive5.com/uparse/).

Total genomic DNA was extracted from water and sediment samples using the E.Z.N.A. Soil DNA Kit (Omega Bio-tek, Norcross, GA, U.S.) according to manufacturer’s instructions. DNA extract was fragmented to an average size of about 400 bp using Covaris M220 (Gene Company Limited, China) for paired-end library construction. Paired-end sequencing was performed on Illumina NovaSeq/Hiseq Xten(Illumina Inc., San Diego, CA, USA) at Majorbio Bio-Pharm Technology Co., Ltd. (Shanghai, China) using NovaSeq Reagent Kits/HiSeq X Reagent Kits according to the manufacturer’s instructions (www.illumina.com).

**Gene prediction**

Open reading frames (ORFs) in contigs were identified using MetaGene (Noguchi et al., 2006) (http://metagene.cb.k.u-tokyo.ac.jp/). The predicted ORFs with length being or over 100 bp were retrieved and translated into amino acid sequences using the NCBI translation table (http://www.ncbi.nlm.nih.gov/Taxonomy/taxonomyhome.html/index.cgi?chapter=tgencodes#SG1.

**Construction of a non-redundant gene catalog** **and** **calculation of gene abundance**

A non-redundant gene catalog was constructed using CD-HIT (Fu et al., 2012) (http://www.bioinformatics.org/cd-hit/, version 4.6.1) with 90% sequence identity and 90% coverage. Reads after quality control were mapped to the non-redundant gene catalog with 95% identity using SOAPaligner (Li et al., 2008) (http://soap.genomics.org.cn/, version 2.21), and gene abundance in each sample were evaluated.

**Analysis of key microorganisms contributing nitrogen and phosphorus genes**

After constructing non-redundant gene catalogs (involving nitrogen and phosphorus cycles), BLASTP (Altschul et al., 1997) (BLAST Version 2.2.28+, http://blast.ncbi.nlm.nih.gov/Blast.cgi, BLAST alignment parameters set expectations for 1e-5) was used to contrast non-redundant gene catalogs and NR Database (Non - Redundant Protein Database). The taxonomy information database corresponding to NR database obtained microorganism (genus level) annotation results. The gene contribution was the percentage of the gene abundance contributed by the microorganism in the total gene abundance. The microorganisms with higher contribution (more than 5%) were defined as the key microorganisms in our study.

**The data analysis tools and methods**

The molecular ecological network analysis (http://ieg4.rccc.ou.edu/mena) based on random matrix theory was used to construct topological networks and reveal potential interactions between taxa in the microbial communities of each sampling zone (Deng et al., 2012). To improve the reliability of the network, we selected only bacterial OTUs that were present in at least 50% of the samples at a site to construct the network. The version 0.9.2 of the Gephi software (https://gephi.org/) was used to perform the network analysis. To extract modules from the networks, we used fast greedy modular optimization (Liu et al., 2019). And the molecular ecological network analysis was used to generate 1000 random networks of equal size, and calculated the average path length (i.e., the average distance between any two nodes in the network), average clustering coefficient (i.e., the degree to which a given node is connected to adjacent nodes), and modularity (i.e., the degree of microbial interaction in a particular microbial community) for the random networks and compared them with the corresponding values for the actual networks. The *t*-tests were applied to determine whether there were significant differences in the network indicators between the observed and random networks. The top 1% of the taxa based on the network degree ranking were identified as the keystone taxa in the topological networks (Hartman et al., 2018). Used Fig Tree v1.4.4 (http://tree.bio.ed.ac.uk/software/FigTree/) and Past4.09 (https://www.nhm.uio.no/english/research/infrastructure/past/index.html) software analyzing microbial samples and ANOSIM difference test. Microbial community structure visualization used circos analysis was performed using the OmicStudio tools (https://www.omicstudio.cn/tool/). The KEGG database (https://www.genome.jp/kegg/) was used to label the nitrogen (KO00910) and phosphorus (KO00440) metabolic pathways. For gene analysis, the corresponding splicing software MetaGene, (http://metagene.cb.k.u-tokyo.ac.jp/), the sequence with the best splicing effect was selected and the Open reading frames (ORFs) was predicted. Genes with nucleic acid lengths greater than or equal to 100bp were selected and translated into amino acid sequences. The Smart PLS 3.0 (Sarstedt and Cheah, 2019) (http://www.smartpls.com.) was used to build a Partial least squares path modelling (PLS-PM) to analyze the influence of environmental factors on key microorganisms/genes and on the concentration of nitrogen and phosphorus nutrients. The goodness of fit (GOF) was used to evaluate the model by the method of Wetzels et al. (2009) and 0.67 indicated a good fit. The significance was tested by bootstrap resampling verification program, and the setting times was 5000 times.

**The topological characteristics of the molecular ecological network**

The *t*-test results showed that major network characteristic parameters of the empirical network (the average path distance, average clustering coefficient, and modularity) were significantly higher than those of the random network (*p* < 0.001). This suggested that the ecological networks were not random structures (Xu et al., 2022). The small-world coefficients of the networks for the four zones were all greater than 1. This result indicates that all networks had small-world characteristics in this study (Humphries and Gurney, 2008); that is, they had high interconnectivity and high efficiency of material and energy transfers (Zhang et al., 2021). In addition, the characteristic value of the network module ranged from 0.635 to 0.728 (**Table 2**), and since they were all higher than 0.4, this suggested that the constructed networks had modular characteristics (Newman, 2006). This shows that the main topological features of the networks were consistent with the general characteristics of molecular ecological networks (Faust and Raes, 2012).

**Supplementary references**

Altschul, S.F., Madden, T.L., Schaffer, A.A., Zhang, J.H., Zhang, Z., Miller, W., et al. (1997). Gapped BLAST and PSI-BLAST: a new generation of protein database search programs. Nucleic Acids Res. 2, 3389-3402. doi:10.1093/nar/25.17.3389.

Deng, Y., Jiang, Y.-H., Yang, Y., He, Z., Luo, F., and Zhou, J. (2012). Molecular ecological network analyses. BMC Bioinform. 13, 113. doi: 10.1186/1471-2105-13-113.

Faust, K., and Raes, J. (2012). Microbial interactions: from networks to models. Nat. Rev. Microbiol. 10, 538-550. doi: 10.1038/nrmicro2832.

Fu, L., Niu, B., Zhu, Z., Wu, S., and Li, W. (2012). CD-HIT: accelerated for clustering the next-generation sequencing data. Bioinformatics. 28, 3150-3152. doi:10.1093/bioinformatics/bts565.

Hartman, K., van der Heijden, M.G.A., Wittwer, R.A., Banerjee, S., Walser, J.-C., and Schlaeppi, K. (2018). Cropping practices manipulate abundance patterns of root and soil microbiome members paving the way to smart farming. Microbiome. 6, 14. doi: 10.1186/s40168-017-0389-9.

Humphries, M.D., and Gurney, K. (2008). Network 'small-world-ness': a quantitative method for determining canonical network equivalence. PLoS One. 3, 0002051. doi: 10.1371/journal.pone.0002051.

Liu, F., Li, Z., Wang, X., Xue, C., Tang, Q., and Li, R.W. (2019). Microbial co-occurrence patterns and keystone species in the gut microbial community of mice in response to stress and chondroitin sulfate disaccharide. Int. J. Mol. Sci. 20, 2030. doi: 10.3390/ijms20092130.

Li, R., Li, Y., Kristiansen, K., and Wang, J. (2008). SOAP: short oligonucleotide alignment program. Bioinformatics. 24, 713-714. doi:10.1093/bioinformatics/btn025.

Newman, M.E.J. (2006). Modularity and community structure in networks. Proc Natl Acad Sci U S A. 103, 8577-8582. doi: 10.1073/pnas.0601602103.

Noguchi, H., Park, J., and Takagi, T. (2006). MetaGene: prokaryotic gene finding from environmental genome shotgun sequences. Nucleic Acids Res. 34, 5623-5630. doi: 10.1093/nar/gkl723.

Sarstedt, M., and Cheah, J.H. (2019). Partial least squares structural equation modeling using SmartPLS: a software review. J. Mark. Anal. 7, 196-202. doi: 10.1057/s41270-019-00058-3.

Wetzels, M., Odekerken-Schroder, G., and van Oppen, C. (2009). Using PLS path modeling for assessing hierarchical construct models: Guidelines and empirical illustration. MIS Q. 33, 177-195. doi: 10.2307/20650284.

Xu, H., Zhao, D., Zeng, J., Mao, Z., Gu, X., and Wu, Q.L. (2022). Evaluating the effects of aquaculture on the freshwater lake from the perspective of plankton communities: the diversity, co-occurrence patterns and their underlying mechanisms. Environ. Pollut. 309, 119741. doi: 10.1016/j.envpol.2022.119741.

Zhang, L., Delgado-Baquerizo, M., Shi, Y., Liu, X., Yang, Y., and Chu, H. (2021). Co-existing water and sediment bacteria are driven by contrasting environmental factors across glacier-fed aquatic systems. Water Res. 198, 117139. doi: 10.1016/j.watres.2021.117139.
